# Aprotic Sulfur–Metal Batteries: Lithium and Beyond

**DOI:** 10.1021/acsenergylett.2c02493

**Published:** 2023-02-06

**Authors:** Daniele Meggiolaro, Marco Agostini, Sergio Brutti

**Affiliations:** † Computational Laboratory for Hybrid/Organic Photovoltaics (CLHYO), Istituto CNR di Scienze e Tecnologie Chimiche (SCITEC-CNR), Via Elce di Sotto 8, 06123 Perugia, Italy; ‡ Dipartimento di Chimica e Tecnologia del Farmaco, Università di Roma La Sapienza, P.le Aldo Moro 5, 00185 Roma, Italy; § Dipartimento di Chimica, Università di Roma La Sapienza, P.le Aldo Moro 5, 00185 Roma, Italy; ∥ Consiglio Nazionale delle Ricerche, Istituto dei Sistemi Complessi, Piazzale Aldo Moro 5, 00185 Roma, Italy; ⊥ GISEL-Centro di Riferimento Nazionale per i Sistemi di Accumulo Elettrochimico di Energia, INSTM via G. Giusti 9, 50121 Firenze, Italy

## Abstract

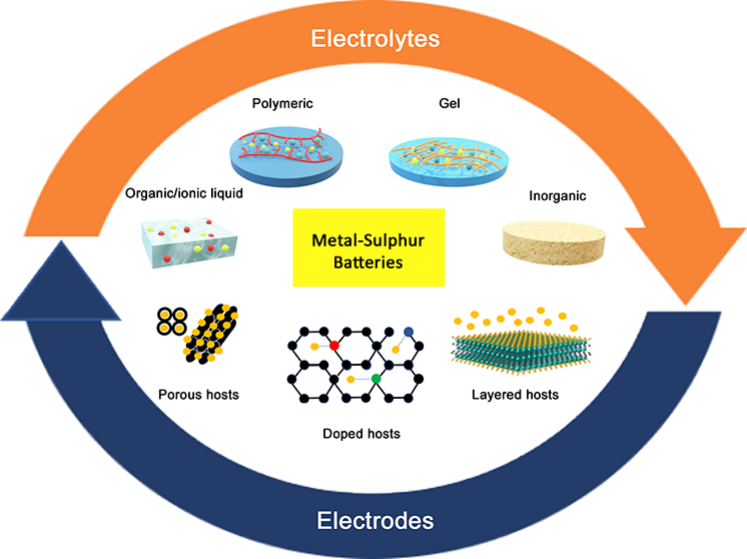

Metal–sulfur batteries constitute an extraordinary research playground that ranges from fundamental science to applied technologies. However, besides the widely explored Li-S system, a remarkable lack of understanding hinders advancements and performance in all other metal–sulfur systems. In fact, similarities and differences make all generalizations highly inconsistent, thus unavoidably suggesting the need for extensive research explorations for each formulation. Here we review critically the most remarkable open challenges that still hinder the full development of metal-S battery formulations, starting from the lithium benchmark and addressing Na, K, Mg, and Ca metal systems. Our aim is to draw an updated picture of the recent efforts in the field and to shed light on the most promising innovation paths that can pave the way to breakthroughs in the fundamental comprehension of these systems or in battery performance.

Since their introduction on the market in the early 1990s, lithium-ion batteries have become the performance benchmark for any further radical improvements in the field of electrochemical energy storage.^1^ Alternative battery chemistries, such as Na-ion,^2^ K-ion,^3,4^ Mg-Ion,^5^ Ca-ion,^6^ Al-ion,^7−9^ and Zn-ion,^10^ as well as the exploitation of lithium metal plating/stripping at the negative electrodes^11^ coupled with intercalation at positive electrodes^12^ or conversion at O_2_ gaseous electrodes,^13^ have been proposed, explored, demonstrated, debated, and in some cases criticized^14,15^ in the past 30 years. An enormous scientific effort is currently in progress worldwide to identify how different technologies can find effective applications in specific niche fields, thus replacing the current ubiquitous Li-ion battery benchmark.^16−19^

In the past 10 years, the exploration of innovative chemistries for the exploitation of sulfur as the positive electrode active material in aprotic batteries has experienced a remarkable boom.^3,7,20−30^ Among all the possible variants, the Li-S formulation is the most advanced one and has been already demonstrated in pre-commercial prototypes.^19,31,32^ There are several advantages in the technological shift from lithium-ion battery chemistry to lithium–sulfur in terms of the volumetric and gravimetric specific energies and specific capacities as well as the costs. The theoretical gravimetric and volumetric specific capacities of a Li-S cell (1167 mAh g^–1^ and 1216 mAh mL^–1^, normalized by the masses and volumes of the active materials at both electrodes) are respectively 11 and 3 times larger compared to the LiCoO_2_/graphite benchmarks or 7 and 2 times larger compared to a hypothetical advanced Li-ion battery formulation of LiFePO_4_/silicon. Furthermore, a qualitative estimate of the costs of active materials in Li-S batteries (Li ∼2.2 € g^–1^; S ∼0.04 € g^–1^)^33^ suggests figures close to the Li-ion formulations (LiCoO_2_ ∼1.3 € g^–1^; LiFePO_4_ ∼1.3 € g^–1^; graphite ∼0.03 € g^–1^; silicon ∼0.34 € g^–1^), thus making the energy stored per € of active materials, i.e., expressed in terms of Wh €_AM_^–1^, much more favorable for the sulfur-based battery chemistry compared to the Li-ion benchmark. This favorable landscape is partially counterbalanced by the remarkable volume variation that affects the Li-S active materials upon charge/discharge, i.e., ∼ –33% of volume upon discharge, compared to the small changes occurring in any Li-ion battery formulation (−8% and −11% on discharge in the LiCoO_2_/graphite and LiFePO_4_/silicon cases, respectively).^21,34^ This huge change in the active materials’ volumes between the charged and discharged states of Li-S batteries unavoidably requires volume buffers inside the electrode architectures,^35,36^ thus reducing the net density, deteriorating the volumetric performance, and increasing the energy cost. Overall, the successful commercialization of Li-S batteries requires finding an optimal balance among performance, long-term calendar life, and cost that is able to overcome the current Li-ion benchmark.

However, Li-S is not the only aprotic sulfur-based battery chemistry currently in the spotlight in fundamental research worldwide: also Na-S, K-S, Mg-S, Ca-S, and Al-S battery chemistries are currently challenging the battery research field.^7,23,25,27,37,38^ To shed some light on this, the same comparative analysis outlined above for the Li-S case can be drawn also in these cases, thus highlighting the comparative merits of each battery chemistry. In Figure 1, the performance features and energy costs per active material mass are compared to the Li-ion and Li-S benchmarks, as well as the maximum volume variation suffered simultaneously by both active materials between charge/discharge. All quantities in the radar plots in panels (a)–(c) are in relative units benchmarked to (*i*) the gravimetric capacity of the Li-S battery chemistry (i.e., 1167 mAh g^–1^ normalized by the sum of the masses of both active materials), (*ii*) the volumetric capacity of the Al-S battery chemistry (i.e., 2484 mAh mL^–1^ normalized by the weighted sum of molar volumes of both active materials), (*iii*) the gravimetric energy of the Li-S battery chemistry (i.e., 2612 mWh g^–1^ normalized by the sum of the masses of both active materials), (*iv*) the volumetric energy of the Ca-S battery chemistry (i.e., 3244 mWh mL^–1^ normalized by the weighted sum of molar volumes of both active materials), and (*v*) the energy stored per € of active materials of the Mg-S battery chemistry (i.e., 16.1 Wh g^–1^ normalized by the estimated cost of the sum of the masses of both active materials).

**Figure 1:**
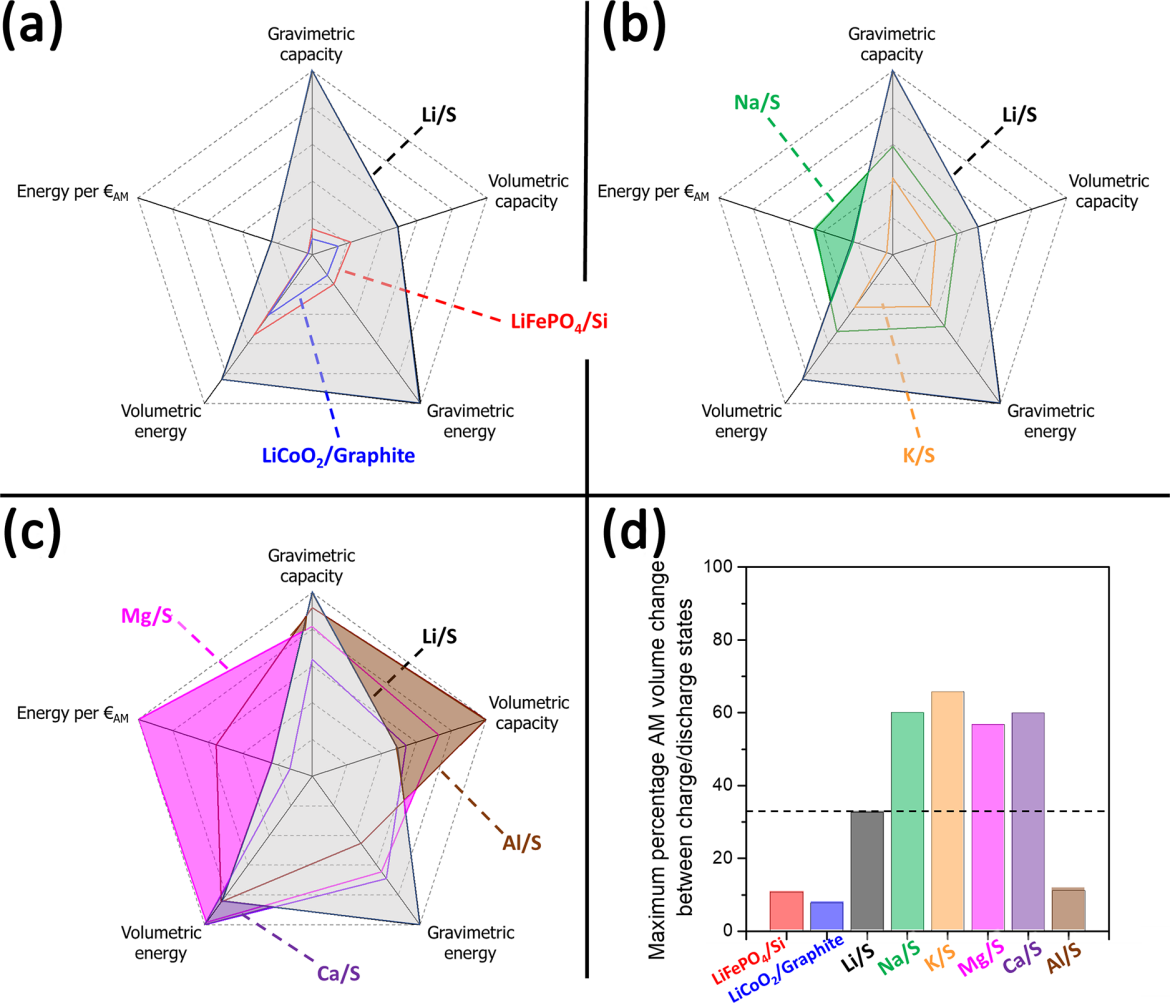
Comparisons among the theoretical features of different competitive battery chemistries. (a) Comparison between the Li-S battery chemistry and two Li-ion benchmarks: LiFePO_4_/Si and LiCoO_2_/graphite. (b) Comparison between the Li-S battery chemistry and the two monovalent ones, Na-S and K-S. (c) Comparison between the Li-S battery chemistry and the three multivalent ones, Mg-S, Ca-S, and Al-S. (d) Comparison of the maximum percentage of combined volume change suffered by both active materials between charge and discharge (the dashed line is the benchmark +33% volume change suffered simultaneously at the positive and negative sides of a Li-S cell in charge).

Overall, all the sulfur-based battery chemistries overcome both Li-ion benchmarks in all the theoretical performance figures and costs, with the only exception being the volumetric energy density of the K-S case. On the contrary, compared to the Li-S benchmark, the landscape is nuanced and requires a case-by-case discussion.

The Li-S battery chemistry largely outperforms both of the monovalent Na-S and K-S ones. However, the lower cost of sodium metal compared to lithium makes the energy stored per € of active materials more favorable in the Na-S battery formulation.

The same applies also for the multivalent Mg-S and Al-S cases, where the theoretical energy stored per € of active materials is respectively 4 and 2 times higher compared to the Li-S case thanks to the lower costs of Mg and Al. Generally speaking, multivalent sulfur–metal chemistries can also provide better theoretical volumetric performance compared to Li-S, thanks to the higher densities of the metals. In fact, the specific volumetric capacities of Al-S, Mg-S, and Ca-S are respectively 2, 1.5, and 1.1 times larger than that of Li-S, whereas the volumetric energy densities of both Ca-S and Mg-S are ∼1.2 times higher than that of Li-S. Overall, multivalent metal–sulfur battery chemistries overcome the Li-S benchmark in both costs and volumetric performance.

On the other hand, all Na-, K-, Mg-, and Ca-S battery chemistries suffer huge overall volume variation between charge/discharge considering both electrodes, as outlined in Figure 1d, approximately doubling the figures of the volume shrinking/expansion in the Li-S case. This is a remarkable drawback, as it necessarily requires the identification of strategies to handle the huge volume variation. On the opposite side, the Al-S case is remarkably better even compared to the Li-S one: apparently the theoretical electrodes’ simultaneous volume variation in discharge is limited to ∼ –11%, a value 3 times smaller with respect to Li-S, thus matching the lithium-ion LiFePO_4_/silicon case. This remarkable feature originates from beneficial opposite trends of the active materials’ densities compared to the other metals and metal sulfides: in fact, Al_2_S_3_ has a lower density compared to all the other sulfides, whereas Al has a higher density compared to all other alkaline or alkaline-earth metals.

Given this promising and intriguing landscape, here we discuss critically the current understandings and technological demonstrations of metal–sulfur battery chemistries beyond the Li-S ones, thus including Na-S, K-S, Mg-S, Ca-S, and the remarkable Al-S.

## Redox Mechanism and Key Challenges

Sulfur-based compounds have been extensively studied as high-capacity positive electrodes to be coupled with metal in metal–sulfur batteries. Although most of the research activity in the field has been focused on Li-based sulfur batteries, sulfur electrodes have also found applications with anodes different from the Li metal, such as others alkali metals (e.g., Na and K) and also multivalent metals (e.g., Ca, Mg, and Al). Generally speaking, an aprotic sulfur–metal battery is constituted by a porous composite positive electrode with a high content of sulfur (or sulfides), an aprotic electrolyte (either solid or liquid), and a negative metal electrode. Different from conventional intercalation/de-intercalation compounds, the electrochemical reaction in metal-S batteries at the positive electrode is the conversion of the elemental sulfur S_8_ to the respective metal sulfide upon reduction, and the opposite on oxidation.

The fundamental theoretical features of the redox chemistry for different metal–sulfur cells are summarized in Table 1, including the theoretical operational voltage and the related gravimetric and volumetric energy densities. The Li-S battery chemistry has the largest gravimetric energy density; however, when considering the volumetric energy density, Ca and Mg outperform lithium, as well as Al. In contrast, Na-S and K-S cell chemistries show the smallest values. Despite the poor theoretical features compared to Li-S, however, the theoretical features of Na-S and K-S also outperform those of commercial Li-ion batteries (see above).

**Table 1:** Comparison of the Fundamental Theoretical Features of Metal–Sulfur Batteries^a^

**Cell type**	**Theoretical redox reaction**	**Thermodynamic*****emf*****(V)**	**Gravimetric energy density(Wh kg**^**–1**^**)**	**Volumetric energy density(Wh L**^**–1**^**)**
Li-S	2Li + S = Li_2_S	2.6	2612	2723
Na-S	2Na + S = Na_2_S	2.3	1262	1680
K-S	2K + S = K_2_S	2.5	914	1149
Mg-S	Mg + S = MgS	1.8	1682	3195
Ca-S	Ca + S = CaS	2.4	1802	3244
Al-S	2Al + 3S = Al_2_S_3_	1.1	1185	2747

a Gravimetric values are calculated with respect to the sum of the metal and sulfur masses.

**Figure 2:**
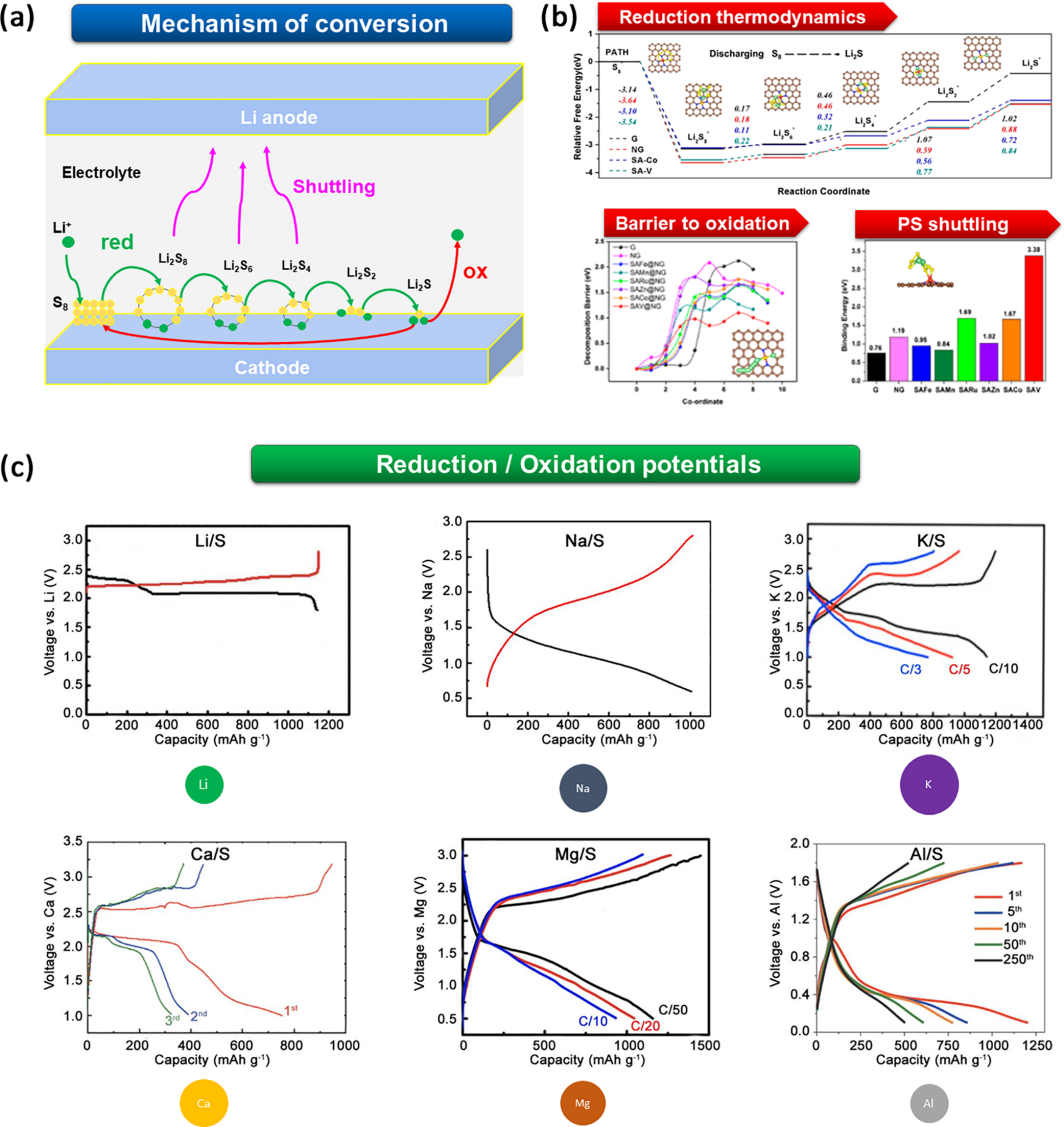
(a) Graphical outline of the conversion mechanism in Li-S batteries. (b) Challenges to the development of metal–sulfur batteries with a focus on Li metal: improve the kinetics of conversion through lowering the energy barrier to polysulfide (PS) reduction and Li_2_S oxidation, and reduce the PS shuttling by increasing the relative adsorption energies at the cathode. [Adapted with permission from ref (41). Copyright 2019 American Chemical Society.] (c) Experimental galvanostatic reduction/oxidation potential profiles of different metal–sulfur batteries. [Li: Adapted with permission from ref (42). Copyright 2015 Royal Society of Chemistry. Na: Adapted with permission from ref (43). Copyright 2016 Elsevier. K: Adapted with permission from ref (44). Copyright 2018 Elsevier. Ca: Reproduced with permission from ref (26). Copyright 2020 The Authors. Published by Wiley-VCH GmbH under Creative Commons license CC-BY 4.0. Mg: Adapted with permission from ref (45). Copyright 2016 American Chemical Society. Al: Adapted with permission from ref (46). Copyright 2019 Wiley.]

A pictorial representation of the most crucial points in the electrochemical reduction/oxidation of sulfur in aprotic metal cells and the positive electrode voltage profiles reported experimentally for all six metals, Li, Na, K, Mg, Ca, and Al, are shown in Figure 2. A detailed review of the most relevant aspects of the redox reactions in Li-S batteries is provided in the Supporting Information (SI); here we focus on the electrochemistry of beyond-lithium metal–sufur cells.

The electrochemical conversion of sulfur using different metallic counter-electrodes shares many similarities with the mechanism proposed in Li-S batteries (see the SI, section “Redox mechanism and key challenges in Li-S batteries”). However, the differences in the ionic sizes and physicochemical properties of the metal cations (e.g., polarizabilities, charge density, overall charge, electronegativity) can affect the reaction path and the kinetics of the process, as outlined qualitatively by the different shapes of the galvanostatic potential profiles shown in Figure 2c. First of all, the thermodynamic stabilities of polysulfides (PSs) are altered by the charge density and polarizability of the positive counterions, and the donor number (DN) of the electrolyte solvent further modifies the stability of different PSs. This interplay leads to different reaction pathways in the reduction/oxidation of sulfur in different metal/S cells. As an example, by using operando UV–vis techniques, the alteration in the thermodynamic stability of PSs with respect to cation size has been demonstrated experimentally, showing that short-chain PSs are more stabilized by large cations (e.g., K^+^) than smaller ones (e.g., Mg^2+^ and Al^3+^).^48,82^ Further proof of this has been obtained by operando XAS, UV–vis, and Raman spectroscopy.^84−86^

Metal cation size also has a strong influence on the stability of various crystalline solid metal sulfides. Indeed, while in Li-S cells Li_2_S is the most stable solid product from cell discharge, Li_2_S_2_ being a metastable product, in K-S cells K_2_S_3_ has a higher thermodynamic stability than K_2_S (−582 kJ/mol vs −410 kJ/mol), which makes the reduction reaction from K_2_S_3_ to K_2_S sluggish.^87^ This picture has been supported by different authors who confirmed the formation of K_2_S_3_ phases at the end of discharge.^88,89^ A similar unexpected mechanism is also observed in Na-S cells, where the stable product of discharge is Na_2_S_2_ instead of Na_2_S.^90^ Differently, Ca, Mg, and Al follow the expected mechanisms, as the stable products at the end of discharge are CaS and MgS^90,91^ and for Al the sesquisulfide Al_2_S_3_.^92^ Focusing on Ca-S, Scafuri and co-workers showed that the reversible conversion of sulfur first to PS species and finally to CaS proceeds at room temperature through the two well-defined plateaus, with a consistent part of the sulfur converted into CaS at the end of the first discharge.^29^

All metal/S battery chemistries share similar drawbacks compared to Li-S: (*i*) PSs are formed and dissolved in the electrolyte; (*ii*) the formation of soluble PSs feeds a “shuttle” effect back and forth to the metal anode, wasting charge; and (*iii*) the precipitation/accumulation of insulating layers is promoted over the metal counter-electrodes. Compared to Li polysulfides (LiPs), Na- and K-PSs (NaPs and KPs) are more soluble in organic solvent-based electrolyte, with consequent higher mobility and a more severe shuttle effect, causing high inefficiency between the discharge/charge process.^93,94^ In contrast, metal–sulfur batteries that exploit multivalent negative electrodes (Mg, Ca, and Al) show limited formation of soluble PSs and a reduced shuttle effect back and forth to the metal anode. In particular, for Ca-S batteries, the occurrence of a shuttle effect has been experimentally excluded by Scafuri and co-workers.^29^ The main reason can be related to the solubility of Mg^2+^, Ca^2+^, and Al^3+^ PSs in organic solvent electrolyte, which is lower than in alkali-based electrolyte, and also reduced mobility of these species.^46,84,96^ Remarkably, the alteration of the electrochemical redox activity of sulfur impacts the reversibility of the metal–sulfur battery: apparently Mg-S, Ca-S, and Al-S cells are all free from the endless charges observed in Li-S, Na-S, and K-S cells without an appropriate passivation film over the metal surface (e.g., promoted by the degradation of sacrificial lithium nitrite).^46,84,93−98^

As they do for Li, density functional theory (DFT) simulations also provide deep insights into the mechanisms of conversion of other metal-S batteries. Simulation of the reduction reactions of Na- and Li-PS species on a V@WSe_2_ host highlighted that the rate-limiting step in the reduction of Na shows a higher barrier to reduction but a lower barrier to oxidation compared to Li, justifying the presence of Na_2_S_2_ as the only kinetic product.^99^ As observed for Li, the use of vanadium (V) as a single-atom catalyst dopant can improve the kinetics in both reduction and oxidation by lowering the barriers of the rate-limiting steps.^99^ Similar effects have been also predicted for the use of a VS_2_ anchoring material that, besides improving Na-PS retention at the cathode, would decrease the thermodynamic barriers to Na-PS reduction to Na_2_S_2_ and Na_2_S and to reoxidation.

DFT stability analysis of Mg-PS in Mg-S batteries predicts a monotonic decrease of the energy of formation moving from long-chain to short-chain Mg-PS (see Figure 2c).^100^ A combined experimental–theoretical work^69^ has demonstrated that the capacity degradation in Mg-S cells with Mg(HMDS)_2_–AlCl_3_ electrolyte is due to the irreversible formation of discharge products, e.g., MgS and Mg_3_S_8_, through a direct electrochemical deposition or a chemical disproportionation of intermediate polysulfides. The same study highlights that an improvement in the kinetics of the oxidation process can be obtained by using TiS_2_ as active electrocatalyst. Moving to Al-S batteries, Bhauriyal et al. provided important details about the charging and discharging processes in Al-S batteries by the analysis of S_8_(001)/[EMIM]AlClS_4_ and Al_2_S_3_(001)/[EMIM]AlClS_4_ interfacial systems by molecular dynamics simulations.^101^ The discharging process can proceed through the continuous reduction of S to Al_2_S_3_-like products via a series of polysulfide intermediate species and involves the formation of various cationic and anionic intermediate species.

## Challenges for the Development of a Reliable Positive Electrode Material

**Figure 3:**
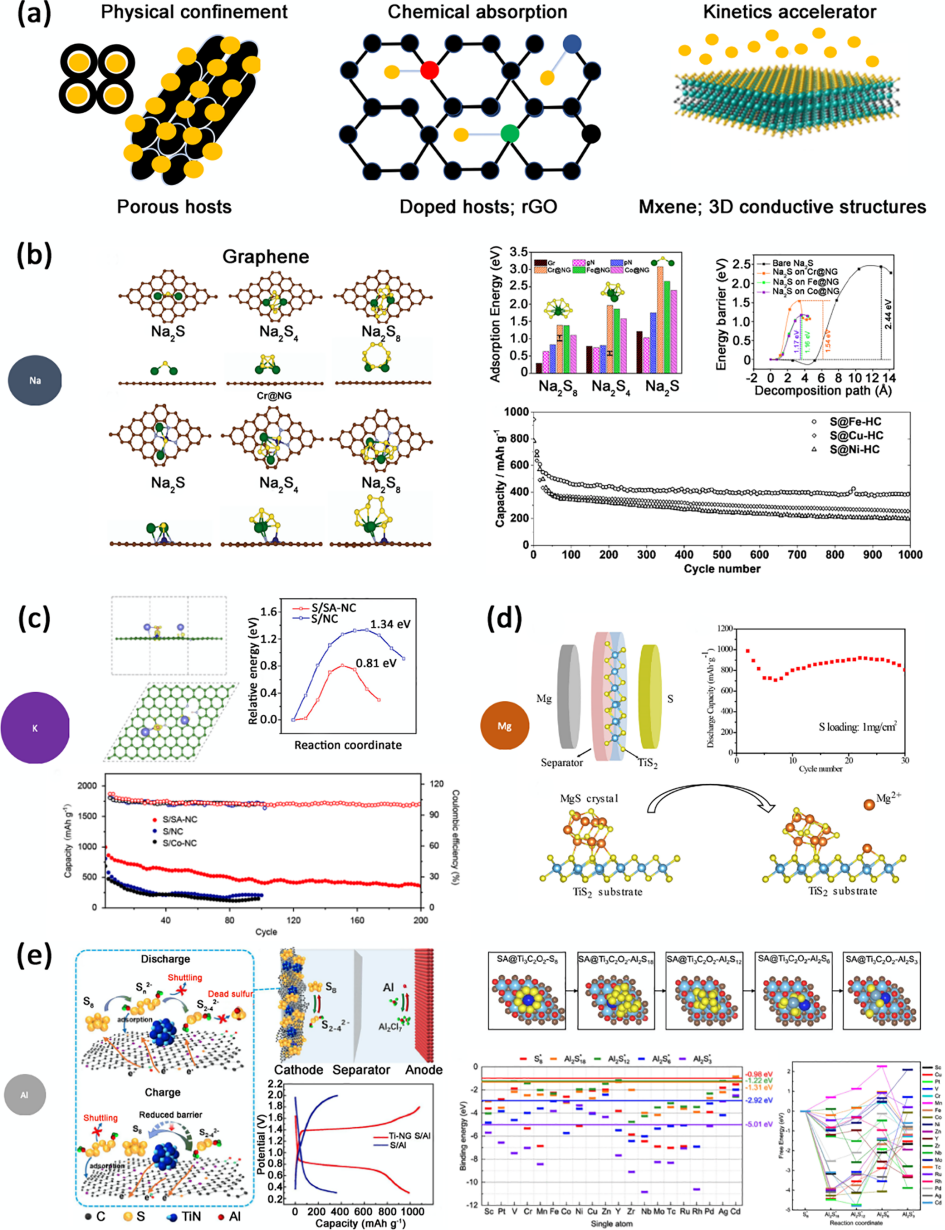
(a) Schemes of the fundamental morphological/structural concepts typically exploited to optimize the performance of a sulfur-composite electrode. (b) Na-S battery: geometric configurations of Na-PS on N-doped graphene (NG) and Cr-SAC NG (Cr@NG); Na-PS adsorption energies on Cr@NG, Fe@NG, and Co@NG; and relative barriers to decomposition. (Adapted with permission from ref (102). Copyright 2021 American Chemical Society.) On the bottom right is shown the cycle performance of a sulfur cathode based on transition-metal (M = Fe, Cu, and Ni) nanoclusters loaded onto hollow carbon (HC) nanospheres. [Adapted with permission from ref (103). Copyright 2019 Wiley.] (c) K-S battery: equilibrium structure, charge difference analysis, and barrier to dissociation of K_2_S on sulfur host based on Co single atoms on N-doped carbon (SA-NC), with the relative cycling performance. [Adapted with permission from ref (104). Copyright 2021 American Chemical Society.] (d) Mg-S battery: schematics of the Mg-S cell with a TiS_2_-coated separator that activates the decomposition of MgS, with the related cycling performance of the Mg-S cell. [Adapted with permission from ref (100). American Chemical Society 2020.] (e) Left: schematics for the working principle of a TiN-NG:S//Al battery and relative charge/discharge profile. [Adapted with permission from ref (105). Copyright 2022 Elsevier.] Right: binding energies of S_8_ and Al_2_S_*n*_ PSs on different SA@Ti_3_C_2_O_2_ nanosheets and relative free energy of conversion. [Adapted with permission from ref (106). Copyright 2021 American Chemical Society.]

Due to its insulating nature and the remarkable volume expansion upon cycling, sulfur is used as an active electrode material in blends with conductive and binder components. The overall goal is to improve the interparticle cohesion between the active material and the conductive particles as well as the adhesion on the Al current collector.^20^ The most common fundamental approaches to rationally draw on paper and manufacture in the lab for sulfur-composite-based electrodes are shown in Figure 3a. The most relevant strategies and successful achievements in the design of innovative and effective positive electrodes for Li-S batteries are broadly reviewed in the SI (section “Challenges for the development of a reliable positive electrode material in Li-sulfur batteries”); in this section we focus on recent advancements to adapt the Li-S concepts to beyond-lithium metal–sulfur formulations.

All beyond-lithium–sulfur battery chemistries face challenges (e.g., PS solubility, shuttle effect) very similar to those already tackled by many research groups for Li-S cells; thus, similar concepts have been exploited to design and demonstrate effective positive electrodes and cell formulations. A carbon-based matrix has been the most used for room-temperature Na-S systems; various morphologies have been proposed to enhance the electron transfer, to facilitate the redox reactions, and to prevent the dissolution of active material and consequent shuttle effect.^146^ Coaxial carbon structures based on microporous carbon sheaths embedded in carbon nanotubes have been designed to accommodate sulfur, however not totally preventing dissolution of Na-PSs in the electrolyte and capacity fading.^147^ The use of hollow carbon (HC) substrate has also being proposed to prevent shuttle effects: however, due to the radical spatial confinement of sulfur, such electrodes fail to exploit the entire capacity of sulfur.^148^

In line with Li-S cells, carbon hosts have been re-designed to promote large sulfur loading and PSs binding by the inclusion of heteroatoms. 3D Ni-HC spheres concatenated in N-doped carbon nanofibers have been proposed to enhance electrochemical kinetics and facilitate Na-PS adsorption on the cathode, due to the formation of Ni–S bonds.^149^ DFT simulations indicating the potential improvements in the conversion kinetics have been also demonstrated in N-doped graphene decorated with single-atom catalyst (Cr, Fe, Co). The presence of the transition-metal catalyst, beyond enhancing the interaction of Na-PS with the carbon matrix, also decreases the barrier to decomposition (see Figure 3b).^102^ Experimentally, this has been demonstrated by the use of transition-metal (Fe, Cu, and Ni) nanoclusters on HC nanospheres, which have been shown to provide good cycling stability through increasing the Na-PS immobilization and activity (see Figure 3b).^103^

Taking advantage of experience with Li-S batteries, metal–organic framework (MOF) networks have also been demonstrated in the design of electrodes suitable for Na-S cells. For example, a zeolitic imidazolate framework, i.e., MOFs ZIF67 and/or ZIF8, can be used to design nitrogen-doped networks for hosting sulfur active material in Na-S cells, reaching a stable capacity of 500 mAh g^–1^ for over 250 cycles.^150^ Polar groups can be used to modify carbon networks and enhance binding energies with Na-PS through polar–polar interactions. For example, carbon nanofibers can be modified using high sulfur catalytic Fe-based species which can lower the Na_2_S oxidation energy barrier, improving its reversibility and consequently the cycling stability towards Na.^151^ Sulfides can also act as polar catalysts in Na-S cells. An example is given by a composite synthesized including MoS_2_ in a nitrogen-doped carbon network, which increased both capacity retention and rate capability, with a Na-S cell able to deliver 360 mAh g^–1^ for over 2800 cycles at 2C rate.^152^

DFT calculations indicate that other sulfide compounds, such as VS_2_ and As_2_S_3_, show satisfactory binding energies vs Na-PS, and they are able to mitigate the shuttle effects.^153,154^ Similarly to sulfides, nitrides such as Fe_2_N have been shown to catalyze the Na-PS conversion.^155^ The chemical and structural synergistic immobilization of Na-PSs in the cathode structure has been realized through the use of aluminum oxyhydroxide (AlOOH) nanosheets decorated with a sulfur/carbon black nanocomposite (S@CB@AlOOH). A coupled experimental–theoretical work indicated that AlOOH catalyzes the redox conversion of the higher-order PSs (Na_2_S_*n*_, 6 ≤ *n* ≤ 8) to the lower-order PSs (Na_2_S_*x*_, 1 ≤ *x* ≤ 2), boosting the performance of the conversion in Na-S batteries.^137^

The use of M-Xene has been also proved in Na-S cells, employing Ti_3_C_2_T_*x*_ nanosheets as sulfur host material. The as-synthesized composite showed reduced delivered capacity with a good cycling stability, while the doping of M-Xene brought about better cycling performance.^141,142^

Both Li-S and Na-S battery chemistries being at the center stage of research in the past 10 years, a great number of electrode configurations have been reported for both systems. DFT calculations have been carried out on double-transition-metal (DTM) MXenes, Mo_2_TiC_2_T_2_ (T = O and S), by analyzing their interactions with S_8_/Na_2_S_*n*_ (*n* = 1, 2, 4, 6, and 8). Both of these materials exhibit moderate Na-PS adsorption energies, and they are expected to effectively inhibit Na_2_S_*n*_ dissolution and shuttling. Furthermore, the calculated Gibbs free energies of the rate-determining step for sulfur reduction and the energy barriers to Na_2_S decomposition are found to be significantly lower than those in a vacuum, suggesting that the use of these MXenes is beneficial in boosting both Na-PS reduction and Na_2_S reoxidation in discharge and charge, respectively.^158^

Differently, research on electrode configurations for K-S or cells with multivalent metals is more recent and in most cases results in applications of electrodes previously developed for Na and Li systems. Mesoporous carbon was used to develop the first K-S cell,^159^ while the analyses were reported of a series of different host materials based on PAN (polyacetonitrile),^160^ microporous carbon,^161^ and carbon nanofibers.^162^ Ye et al. reported a combined experimental–theoretical work on K_2_S oxidation on a sulfur host with Co single atoms (Co-SAC) immobilized on nitrogen-doped carbon (NC). Apparently, a synergistic beneficial effect originates from Co-S and N-K lateral interactions and promotes the catalysis of the K_2_S oxidation. Analysis of the barrier to decomposition calculated by DFT shows that Co-SAC has a reduced transition-state energy, significantly lower than that for NC, consistent with the better cycling stability observed experimentally (see Figure 3c).^104^ As for Li and Na, researchers started to apply hosts with higher electronic conductivity, for example, those based on MXene;^163^ however, sulfur-host-based graphene, oxide, and MOF networks have yet to be applied in K-S systems.

Mg-S positive electrodes are typically based on open-scaffold hosts with different porosity and surface area, like activated carbon cloth,^164^ MOFs,^165^ carbon black,^166^ and graphene.^167^ As found with other metal–sulfur batteries, nitrogen doping of carbon has been shown to be an effective strategy to increase Mg-PS immobilization at the cathode and to improve the sluggish kinetics of reduction.^28^ Similarly, M-Xene-based host architectures, i.e., Co_3_S_4_@MXene and CoO-MXene, have been shown to increase the Mg-PS retention by providing good Mg-ion mobility with consequent beneficial effects on the reduction kinetics.^169^ Mo_*x*_S_*y*_ has been also used in Mg-S cells,^170^ as well as TiS_2_, that proved to be able to activate the conversion of low-order MgS_*x*_ and MgS, supplying up to 900 mAh·g^–1^ with good cycling retention (see Figure 3d).^100^ More generally, the concept of the use of functionalized layers of separators to bind dispersed PS has been proposed and demonstrated for Li-S cells,^171^ and it is surely a valid strategy that can be applied to all metal–sulfur formulations.

Turning to Ca-S cells, due to the lack of a reliable electrolyte, the entire mechanism and positive electrode chemistry are less understood; apparently both the shuttle effect and PS dissolution are less pronounced compared to those in Li, Na, and K systems. As far as we know, all reports about Ca-S cells have exploited simple sulfur–carbon composites at the positive side by applying mesoporous carbon materials.^37^ On the contrary, in the case of Al-S systems, where the shuttle effect of PS is remarkable, the majority of publications have exploited sulfur host structures based, for example, on activated carbon,^84^ MOFs,^46^ M-Xenes,^106^ and doped graphene.^105^ Ai et al. developed a TiN@N-doped graphene catalyst for use as the sulfur cathode in Al-S batteries, which suppressed the Al-PS shuttle effect, improved the redox kinetics, and reduced the decomposition barrier, being able to deliver ∼993 mAh g^–1^ in the first cycle and to maintain a capacity of ∼500 mAh g^–1^ after 200 cycles (see Figure 3e).^105^ On the computational side, an extensive DFT study was carried out by Wang et al. in order to unravel the anchoring properties of SAC-decorated (SA@Ti_3_C_2_O_2_) M-Xenes by screening several SAC metals. Their analysis showed that SAC = Y, Nb, Mo, and Tc are potential candidates for high-performance cathodes, showing good adsorption energies for Al-PS and low reaction barriers (see Figure 3e).^106^

## Towards a Suitable Electrolyte for Reversible Sulfur Electrochemistry

As discussed in the previous sections and in the SI for Li-S cells, extensive efforts have been devoted to the design of positive electrode host matrices able to suppress the metal-PS species in the electrolyte through the addition of doped-carbon and polar materials with strong binding energies. Another approach to suppress metal-PS shuttling is the development of electrolytes able to suppress metal-PS dissolution based on tailored liquid solutions, polymer membranes, or inorganic ceramic ionic conductors. However, despite the good qualitative comprehension of the fundamental mechanism of PS dissolution, the fundamental thermodynamics and kinetics of this bundle of processes are still unknown. Particularly, the solubility of many PSs in many solvents is not known, partly due to the difficulty to isolate them, and their disproportionation and interconversion are only poorly outlined in the literature.

Overall, the key points to understand the behavior of PSs within the electrolyte are (*i*) the way PS intermediates interact with the solvent molecules and the metal-ion salt, (*ii*) the effect of salt addition on solvation structure and dynamics, and (*iii*) the effect of PS chain length on the structure and dynamics.

Contrary to Li-S batteries, in Na-S batteries long-chain PS dianions (*n* > 4) are thermally unstable and PS mainly exists as radical monoanions, small dianions, and ion pairs (*n* = 2 and 3). According to DFT calculations, the primary reduction product of S_8_ is the radical anion, which decomposes at the operating temperature of Na-S batteries exoergonically to S_2_^–^ and S_3_^–^ radicals together with the neutral species S_6_ and S_5_, respectively. The S_8_^–^ radical is predicted to disproportionate to S_8_ and S_8_^2–^, followed by the dissociation to two S_4_^–^ radicals. By recombination reactions, these species further interact and react. However, PS dianions larger than S_4_^2–^ are thermally unstable at 320 °C, and smaller dianions as well as radical monoanions dominate in Na_2_S_*n*_ (*n* = 2–5) melts instead.^183^

In line with the fundamental understanding of the role played by the liquid electrolyte in aprotic liquid metal–sulfur batteries, from the experimental point of view three major concepts of electrolytes have been developed—sparingly solvating electrolyte (SSE), moderating solvating electrolyte (MSE), and highly solvating electrolyte (HSE)—that can suppress and promote PS conversion.^184^ A compact review of the most relevant achievements to develop effective electrolytes for Li-S cells is given in the SI (section “Towards a suitable electrolyte for reversible Li-sulfur electrochemistry”).

Moving to Na-S cells, electrolytes are mainly designed closely matching those used in Li-S cells. Typical sodium salts used are NaClO_4_, NaCF_3_SO_3_, and NaPF_6_, while organic solvents are TEGDME, DOL/DME 1:1 mixture, and others glymes. As already discussed, the most relevant drawback of the use of liquid electrolytes with ethereal solvents is the high solubility of Na-PS. As a consequence, in these electrolytes, the electrochemical kinetics of sulfur is maximized as well as the impact of the shuttle effect of NaPS, with consequent poor reversibility.^190^ Similar to Li-S cells, also for Na-S, additives to the electrolytes have been proposed to mitigate Na-PS shuttling. In one case NaNO_3_ was investigated but did not have the prolonged and beneficial effect of LiNO_3_ in Li-S cells.^191^ Ionic-liquid-based liquid electrolytes have also been proposed for Na-S cells and showed good performance and cycling stability, likely due to the poor Na-PS solubility in highly ionic media.^192^

Regarding hybrid configurations, gel polymer electrolytes have been investigated in different configurations, including NaCF_3_SO_3_ and/or NaClO_4_ dissolved in ether-based electrolyte and embedded in polyacrylonitrile nanofibers, poly(ethylene oxide), poly(vinylidene difluoride), and Nafion membrane.^193−195^ These electrolyte configurations can achieve an ionic conductivity of 10^–4^ or 10^–3^ S cm^–1^ at room temperature with an unavoidable loss in the overall Na-S cell performance. On the other hand, the shuttle effect and Na-PS solubility are strongly reduced.

**Figure 4:**
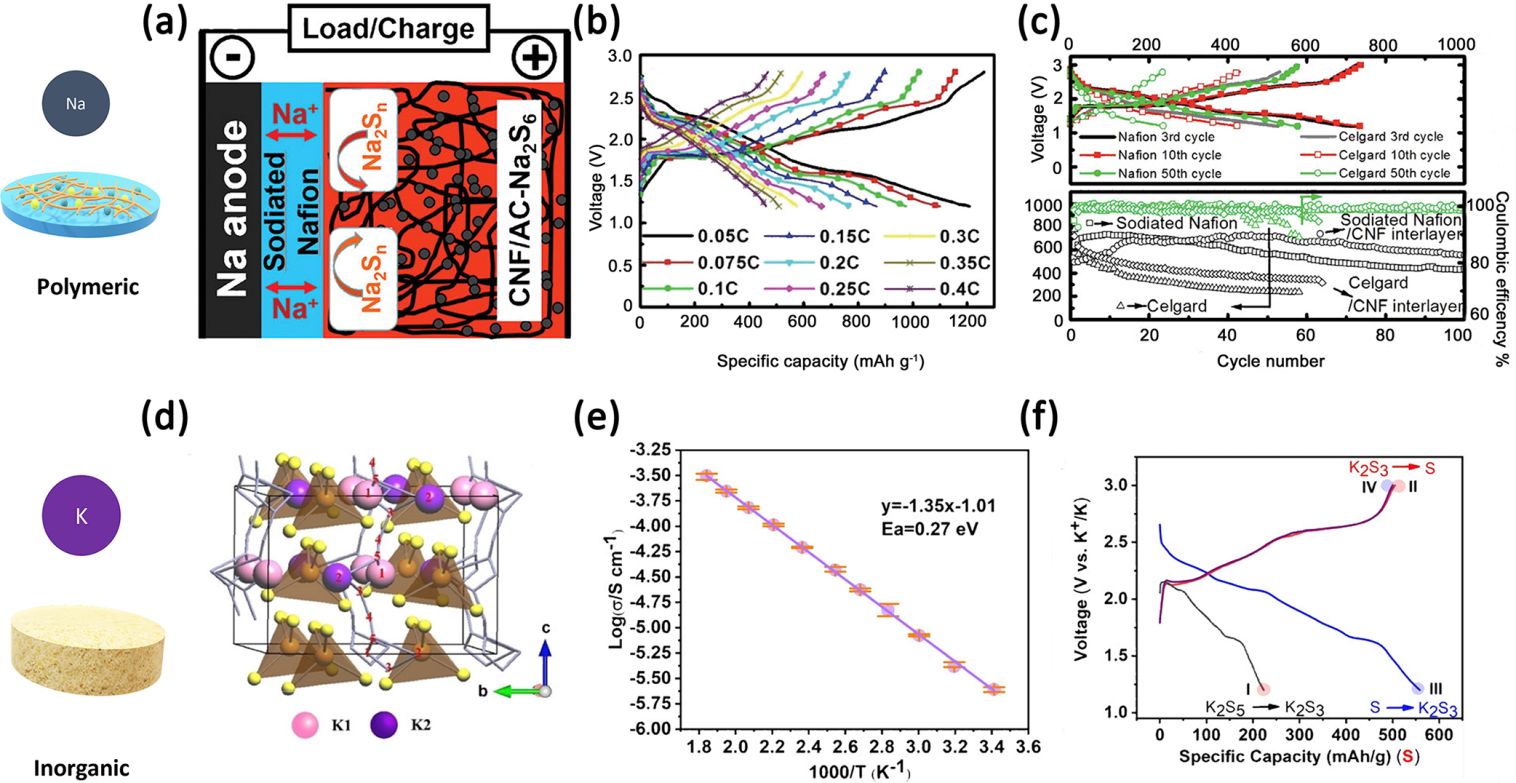
(a) Schematic representation of a Na-S cell using a Nafion/polymer-based electrolyte and (b, c) the corresponding electrochemical performance. [Adapted with permission from ref (193). Copyright 2015 Wiley.] (d) Pictorial scheme of the structure of a potassium superionic conductor and (e, f) its experimental validation as a solid-state ceramic electrolyte in K-S batteries. [Adapted with permission from ref (24). Copyright 2022 Wiley.]

Turning to solid-state-based electrolytes, polymer-based systems have been demonstrated in metal-S batteries beyond lithium; examples of the use of polymeric electrolytes for Na-S batteries are shown in Figure 4a–c. Nafion-based electrolytes apparently allow a good reversibility of the Na-S conversion reactions (see Figure 4b). Voltage profiles are remarkably altered compared to those with liquid electrolytes (see Figure 2c) as the conversion mechanism in solid electrolytes mitigates the formation of long-chain polysulfides, thus leading to improvements in the delivered capacity compared to that in liquid electrolytes (see Figure 4c).^193^

Solid-state room-temperature Na-S cells have also been demonstrated using ceramic electrolytes; similarly to the case of polymeric electrolytes, suppression of the Na-PS solubility enhances the reversibility but the unavoidable small conductivity hinders good rate performance.^197−199^ Overall the optimal configuration in solid-state electrolyte is obtained with a Na-salt with a low lattice energy and a polymer with high dielectric constant to improve its dissociation and the transport of solvated Na^+^ cations; in this way Na^+^ cations are poorly bound to counterions while polymers’ polar groups weakly coordinate both anions and cations. Furthermore, the use of polymers offers advantages of flexibility, good resistance in electrode volume during cycling, shape modulation, and good interfacial contact. A major disadvantage is the low electronic conductivity at room temperature that requires increasing the operational temperature, at least 60 °C, or increasing the amorphous phase in the polymeric matrix.^197^

Turning next to K-S electrochemistry, the research efforts still being at early stages, all the proposed liquid electrolyte concepts are still derived from the other Li-S and Na-S cell chemistries.^25,88,89,160^

Very recently a solid-state electrolyte able to prevent K-dendrite formation has been proposed based on a W-doped K_3_SbS_4_ superionic conductor (see Figure 4d–f). The high conductivity of 1.4 × 10^–4^ S cm^–1^ at 40 °C, among the best reported in the state-of-the-art, and the advantage of solid-state electrolyte, blocking both K-dendrite growth and PS shuttle, demonstrated the possibility of good cycling performance.^199^

Differently from the alkali metal–sulfur cells, the traditional Grignard reagents and conventional magnesium ion electrolytes are nucleophilic and not compatible with sulfur; thus, their design cannot follow those reported for non-aqueous Mg-ion batteries. In this respect the major drawback to building a stable Mg-S cell lies in the difficult task of designing an appropriate electrolyte. The first rechargeable Mg-S cell was reported in 2011 with the design of a non-nucleophilic electrolyte, synthesized with AlCl_3_ and hexamethyldisilazide magnesium chloride (HMDSMgCl).^166^ Other more recent non-nucleophilic Mg-electrolytes are still under development^201,202^ but in most cases are still facing the unsatisfactory reversibility of the Mg plating/stripping, and therefore their application to sulfur electrodes is premature.

In line with magnesium, also the Ca-S electrochemistry faces the lack of a reliable electrolyte for the Ca plating/stripping. A first reported example of Ca-S electrolyte involved dissolving Ca(ClO_4_)_2_ salt in CH_3_CN solvent. Despite delivering a good capacity, the Ca-S cell suffered from the continuous growth of a passivation layer on the Ca surface, reducing the cycle life. This drawback has been partially addressed recently by Manthiram using a dual-cation electrolyte with lithium ions.^37,91^ Very recently, new complex electrolytes have been demonstrated for the plating/stripping of calcium metal in aprotic media at room temperature; hopefully, new experimental validations of these formulations in Ca-S cells will soon appear in the relevant literature.^26,204^

In passing it is important to mention that the development of either Mg-S or Ca-S battery formulations requires tackling the facile tendency towards the formation of ionically non-conductive anodic passivation layers; this is a major factor that can restrict electrolyte selection and optimization for both systems.

Electrolytes are also a key challenge in Al-S batteries, as the electrolytes designed for Li and Na based on organic solvents are not suitable. A first example of an Al-S cell was reported in 2016 based on ionic-liquid electrolyte, i.e., 1-ethyl-3-methylimidazolium chloride ([EMI]Cl) and aluminum chloride (AlCl_3_), however with poor cycling performance.^205^ Generally speaking, there is a relevant research activity towards the identification of novel electrolytes for Al-ion batteries.^206^ It is likely that these innovative formulations will find applications in Al-S cells soon.

## Metal–Sulfur Batteries: A Summary

The theoretical figures of the performance of metal–sulfur batteries are extraordinary and promise the possiblity to develop a variety of innovative battery chemistries, plausibly adapted to the requirements for different applications. However, besides the largely explored Li-S system, a remarkable lack of understanding hinders advancement and effective performance demonstration in all metal–sulfur systems. In fact, numerous similarities and differences make all generalizations in metal–sulfur batteries highly inconsistent, thus suggesting the unavoidable need for extensive research exploration specific for each formulation.

Overall, general concepts are still valid, and the most relevant challenges are similar:

1. the development of a composite electrode with high sulfur loading, optimized buffer volumes, and excellent electronic conductivity;
2. the limitation of metallic PSs in the electrolyte; and
3. the protection of the metal surface by a strong limitation of the shuttle effect.

Tuning the electrode meso-morphologies as well as the surface composition and moieties is a key strategy to simultaneously tackle all these challenges.

On the other hand, the impact of each of these open problems is different in the different metal–sulfur formulations, in particular due to the different thermodynamic landscape. In fact, molecular polysulfide stability and solubility compete with the clusterization driving forces as well as with the thermodynamic stability of the crystalline polysulfide. All these thermodynamic features are unavoidably strongly altered by the use of different metal counterions, with different polarizability, size, and charge density. Therefore, the development of suitable innovative positive electrodes and electrolytes for metal–sulfur batteries beyond lithium is still based on random explorations or serendipity-driven intuition rather than a rational understating of the thermodynamic and kinetic fundamentals of these multinary systems. In particular a clear comparative study of the thermodynamics of reduction/oxidation of multi-phase systems with different metal–sulfur compositions is missing, based either on purely macroscopic thermodynamic modeling or on microscopic ab initio methods. This lack of understanding is further weakened by the unavailability of reliable models available to mimic the dynamics and the reactivity of electrolyte/sulfur and metal/electrolyte interfaces in multi-phase systems like in any metal–sulfur battery. In this respect, wide and systematic computational and experimental research efforts are strongly needed to shed light on and rationalize the reactivity of the different metal–sulfur systems at the positive electrode side as well as in the electrolyte.

As a last point of discussion, it is important to underline that the success of these battery paradigms based on metal negative electrodes also requires a highly reversible metal plating/stripping process, in terms of almost unitary electrochemical and chemical yields as well as in terms of good preservation of the metal morphologies. In all metal–sulfur battery chemistries, this last topic is marginally studied in comparison to positive electrodes and electrolytes, as well as the analysis of formation cycles, gas release, thermal/electrical/mechanical abuse, and realistic cycle-life assessments. Thus, metal–sulfur batteries constitute an extraordinary research playground that ranges from fundamental science to applied innovations.

## Supporting Information Available

Additional review sections: “Redox mechanism and key challenges in Li-S batteries”, “Challenges for the development of a reliable positive electrode material in Li-sulfur batteries”, and “Towards a suitable electrolyte for reversible Li-sulfur electrochemistry” (PDF)
