# Supplementary material for: Aprotic Sulfur–Metal Batteries: Lithium and Beyond
Source: ACS Energy Lett. 2023 Feb 6;8(3):1300–12. doi: 10.1021/acsenergylett.2c02493 (PMC10012267; doi:10.1021/acsenergylett.2c02493)
Supplement: Supplementary file 1 — nz2c02493_si_001.pdf [file nz2c02493_si_001.pdf]

Supplementary information

Aprotic Sulfur-Metal batteries: lithium and beyond

Daniele Meggiolaro,[1,] Marco Agostini[2,]* and Sergio Brutti[3,4,5,]**

[1] Computational Laboratory for Hybrid/Organic Photovoltaics (CLHYO), Istituto CNR di Scienze e Tecnologie Chimiche (SCITEC-CNR), Via Elce di Sotto 8, 06123, Perugia, Italy.

[2] Dipartimento di Chimica e Tecnologia del Farmaco, Università di Roma La Sapienza, P.le Aldo Moro 5, 00185 Roma (Italia)

[3] Dipartimento di Chimica, Università di Roma La Sapienza, P.le Aldo Moro 5, 00185 Roma (Italia)

[4] Consiglio Nazionale delle Ricerche, Istituto dei Sistemi Complessi, Piazzale Aldo Moro 5, 00185 Roma (Italia)

[5] GISEL-Centro di Riferimento Nazionale per i Sistemi di Accumulo Elettrochimico di Energia, INSTM via G. Giusti 9, 50121 Firenze (Italia)

Corresponding Author

* All authors contributed equally to this paper. Corresponding authors – e-mails: sergio.brutti@uniroma1.it; daniele.meggiolaro@cnr.it; marco.agostini@uniroma1.it

Redox mechanism and key challenges in Li-S batteries

The mechanism of conversion at the cathode in Li-S cells has been widely studied. A first attempt to rationalize the conversion process was reported in the early 1979 by Abraham et al., that originally proposed a Li/S cell able to be discharged in organic electrolyte with an electrochemical mechanism based on the formation of different Li_2S_n polysulphides (PS).[1]

Following, in 1988 Peled et al. investigated the oxidation/reaction mechanism of such PS in the same organic solvent through the use of cyclic voltammetry technique, speculating on formation of Li_2S_n with different chain length and on a disproportionation reaction upon reduction process of such PS to form final solid and insoluble Li_2S/Li_2S_2 phase.[2] These studies contributed to unveil the conversion mechanism of S_8 in Li/S cells, that is a complex redox reaction involving a multi-step reduction of sulfur with the formation of soluble PS intermediates in different oxidation states (Li_2S_n, $2<n<8$)

$$2e^- + 2Li^+ + S_8 = Li_2S_8\ (+\ 2e^-) = Li_2S_6\ (+\ 2e^-) = Li_2S_4\ (+\ 2e^-) = Li_2S_2\ (+\ 2e^-) = Li_2S$$

with a liquid-solid phase transition to form Li_2S_2 and Li_2S species in the last reduction step (see Figure 2a, main article). The opposite process occurs on oxidation with the conversion of solid Li_2S/Li_2S_2 to the PS and elemental S_8.

The complexity of the process is increased by the coexistence of disproportionation reactions for S_8^{2-} and S_4^{2-} and by the soluble nature of long-chain Li-PS in the electrolyte. Despite the high theoretical capacity provided by the process, several factors limit its efficiency:

(i) the insulating nature of the sulfur active materials and discharge products, hindering the charge transfer at the interface.

(ii) the sluggish kinetics of conversion due to the presence of thermodynamic barriers to reduction and oxidation.

(iii) the soluble nature of Li-PS which are shuttled back and forth at the electrodes, with possible precipitation at the anode side and consequent loss of active material, i.e. the shuttle effect;[3–6]

(iv) the occurrence of PS-activated side reactions at the anode side.

(v) the large volume expansion at the cathode upon conversion.

To partially overcome some of these issues, sulfur active material is dispersed in host matrices, mainly porous carbon-based compounds and transition metal polar material, such as oxides, carbides, sulphides, nitrides, (see the section "Challenges for the development of a reliable positive electrode material" in the main article), in order to improve the electrical contact at the interface and confine Li-PS at the cathode. Large effort is currently devoted to the development of more performant cathode materials and suitable electrolytes able to limit these drawbacks and to improve the efficiency of the conversion process. For this, a detailed comprehension of the mechanism of conversion and the factors limiting its efficiency is fundamental.

Details about the mechanism of electrochemical conversion in Li/S batteries have been provided by the combination of several experimental techniques and density functional theory (DFT) calculations. High performance liquid chromatography and UV-vis absorption were used to investigate the Li/S reduction mechanism in tetraglyme-based electrolyte.[7] The assessed mechanism upon reduction is based on three steps along the lithium loading potential profile (see figure 2c, main article):

(i) formation of long chain PS during the first reduction step (2.4-2.2 V vs. Li^+/Li) including also the formation of S_3^{*-} radical;

(ii) reduction to S_4^{2-} during the second reduction step (2.15-2.1V vs. Li^+/Li), leading to the gradual decrease of the PS chain lengths;

(iii) reduction to short PS in the final stage of the reaction (2.1-1.9 V vs. Li^+/Li).

From the experimental point of view the reaction kinetics and reaction mechanism of Li/S redox reaction in liquid electrolytes has been further explored by the rotating-ring disc electrode technique confirming the fast reduction of sulfur in the first step of discharge, i.e. during the formation of long chain PS, followed by a slow step of complete conversion into Li_2S accomplished via chemical PS recombination/dissociation reaction.[8] Besides pure electrochemical or ex situ studies, the intensive use of operando techniques has been disclosing very accurate description of the conversion mechanism in Li-S batteries, decoupling the impact of the cell formulation, either at the cathode or electrolyte level. Operando Raman spectroscopy studies shows the formation of long chain (S_8^{2-}) PS via opening of S_8 ring in the first reduction process, at 2.4 V vs Li^+/Li, and short chain PS (S_4^{2-}, S_4^{-}, S_3^{*-}) and Li_2S_2 in the second reduction process at 2.3 V vs Li^+/Li.[9] whereas the impact of different organic electrolyte solutions on the mechanism has been investigated by UV/Vis operando spectroscopy proving the remarkable impact of dielectric constants on the 1 e^- reaction sequence.[10]

Turning to the oxidation step, operando Raman spectroscopy shows that the rapid formation and migration of shorter chain PS is apparently able to delay the formation of long chain PS, thus improving the net utilization of sulfur.[11] This evidence has been consolidated by operando X-ray diffraction, X-ray microscopy and X-ray micro-tomography studies that analysed the dependence of sulfur recrystallization on the preparation technique of the cathode,[6] and the remarkable special inhomogeneities in the chemical speciation across the Li-S cells observed during operation. [12] These accurate spatially-and-time resolved experiments further demonstrate the accumulation upon cycling

of Li_2S on the porous structure of the positive electrode to form a continuous and insulating layer, thus highlighting one of the most detrimental processes that hinders the reversibility of Li/S cells.[13–15]

A better understanding the Li/S reaction mechanism has been also obtained by investigating the species formed at the negative electrode side over lithium metal in the so-called solid electrolyte interface (SEI), through the use of X-ray photoelectron spectroscopy.[16,17] In particular the SEI formed using $LiNO_3$ containing electrolytes hinders the shuttle effect in presence of solubilized PS thus suggesting practical strategies to extend the calendar life of Li-S batteries circumventing the thermodynamic constraints.[18–20]

The rationalization of the thermodynamic and kinetic foundation of the sulfur conversion reaction in Li/S cells has been consolidated by a remarkable computational research effort mainly using DFT approaches.[21,22] The slow kinetics of conversion in Li/S batteries mainly stems from the existence of thermodynamic barriers to the full conversion of long-chain Li-PS to Li_2S_2 and Li_2S upon reduction and to the decomposition of the Li-S bond in discharge products, dominating the re-oxidation process (see the Figure 2b, main article).[23–25] These drawbacks increase the cell over-potential by simultaneously boosting the dissolution of Li-PS in the electrolyte. On the other hand, the kinetics can be modulated by using specific substrates including single atom catalysts (commonly transition metals) embedded in carbon matrices or polar materials, e.g. oxides, nitrides, carbides, sulphides.[21,22,26] This is illustrated by the analysis of the free energies of conversion of the different Li-PS discharge intermediates in pristine and doped graphene (see Figure 2b, main article).[27,28] After the initial exothermic reduction of S_8 to form Li_2S_8, all the subsequent four steps forming Li_2S_6, Li_2S_4, Li_2S_2, and Li_2S are either endothermic or nearly thermoneutral. In particular, the last two steps leading to the formation of Li_2S_2 and Li_2S shows the largest free energy variations and represent the rate

limiting steps of the process. Upon oxidation the rupture of the Li-S bond in Li_2S to form Li-PS is the rate limiting step, by showing barrier to decomposition of ~2 eV in pristine graphene (see Figure 2b, main article). The inclusion of Co and V, as single atom catalysts, reduces the rate limiting steps barriers both in reduction and oxidation compared to N-doped graphene, with beneficial impact on the kinetics of the process. With a similar approach, the superior catalytic activity of other TM catalysts and polar substrates have been demonstrated.[28–31]

Although the kinetics of the conversion can be boosted using substrates with enhanced catalytic activity, another key factor driving the efficiency of the conversion mechanism is the formation of a stable bond between Li-PS and the positive electrode, to improve the electron transfer and to limit the dissolution of metal-PS into the electrolyte. DFT studies have been carried out to analyze the factors favoring the adsorption of the different Li-PS intermediates (Li_2S_x, x=1-8) on routine host materials, including pristine and doped nano-carbon materials, metal oxides, nitrides, carbides and sulfides.[25,26] Due to their large surface area, lightweight and good conductivities, carbonaceous materials have been largely explored as anchoring materials in Li/S batteries.[32,33] The analysis of the binding energies of Li-PS on bare and doped carbon substrates highlights that Li-PS retention is favored on polar surfaces and by the presence of dopant ions with lone pairs, favoring the formation of dative bond with Li.[34] As a results, non-polar carbon substrates show only moderate adsorption energies, while doping with aliovalent ions shows to increase Li-PS adsorption energies compared to bare carbon nanostructures (see also Figure 2b, main article).[35–37] Li-PS interaction at the surface of polar materials, such as nitrides and sulphides, has a more complex trend, being strongly influenced by the termination of the host surface. The computational analysis carried out by Zhang et al. highlighted that Li-PS adsorption on polar host surfaces mainly derives from a charge transfer between the d-orbital of the metal and p-orbitals of S.[38] Interestingly, the analysis of the trend among

different host metals shows that the interaction increases by increasing the number of d electrons in the metal.[38]

The kinetics of conversion is also influenced by the diffusion capability of reactive intermediates, avoiding their accumulation as inactive species at the cathode surface. As shown by Tao et al. with DFT-nudged elastic band (NEB) calculations, the migration barriers of Li at the surface of oxide hosts are strongly influenced by the nature of the metal.[39] This suggests that the kinetics of the conversion process may be modulated by an appropriate choice of the non-conductive hosts, as shown for MgO where the predicted low barriers of migration are consistent with its excellent cycling stability.[39]

Challenges for the development of a reliable positive electrode material in Li-sulfur batteries

The early tries to develop host materials for Li-sulfur batteries positive electrodes have been exploiting the use of non-polar conductive materials, such as those based on mesoporous carbon, where S-particles are "dispersed".[40] These designs showed remarkable limitations, such as the reduction in Li/S cell capacity due to the large amount of redox inactive species (carbon, Al-current collector and polymer binders). Furthermore, being highly dispersed in the mesoporous carbon, part of the sulfur resulted electronically isolated and thus unable to react further decreasing the delivered specific capacity at acceptable cycling rate.[31] An additional detrimental aspect is the hydrophobicity of the carbon hosts, while metal PS species are hydrophilic. As consequence metal-PS formed during the reduction/oxidation process are unavoidably poorly bonded to the carbon host and are prone to the dissolution in the electrolyte.[41]

These drawbacks pushed a radical re-design of the host material by including polar groups at its surface, in order to improve the binding energy with metal PS. The doping of carbon with

electronegative heteroatoms, such as N or O, has been explored by many authors, unfortunately leading to poor electronic conductivities.[42,43] In parallel graphene-based nanomaterials have been explored in both reduced and oxidized forms: apparently the presence of oxygen improves interactions with metal PSs[44] without detrimental effects on the electronic conductivity.[45]

The in-silico design of more efficient carbon-based cathodes proceeded in parallel with experiments.[26] As already mentioned the major drawback of carbon hosts is the limited Li-PS retention at the surface due to their non-polarity, resulting in a pure physical interaction at the interface. DFT-based works intensively focused on heteroatom doping strategies to improve the chemical interaction between Li and the carbon matrix. A systematic study carried out by Hou et al. analysed the impact of several dopants, i.e. N, O, B, F, S, P, and Cl on the adsorption energies of Li-PS in graphene, by furnishing important design principles of the cathode host.[34] Results show that strategies based on N and O doping are effectively able to improve the binding energies of Li-PS in graphene, in agreement with previous findings.[46–49] On the other hand, doping with B, F, S, P, and Cl is less effective to enhance the Li-PS retention, even though other studies highlighted that doping with B and S can effectively increase Li-PS binding energies through an enhanced interaction between B/S and S of the Li-PS.[50,51] Multiple doping strategies, combining the synergistic effects of two or more dopants, have also been also studied.

The combination of N and S doping has been shown to greatly enhance Li-PS adsorption in carbon support due to the beneficial competition of N and S dopant sites to anchor PSs, thus improving the charge transfer kinetics and a better immobilization of PS ions.[52–55] Similar effects have been also observed in N-P co-doped graphene[56] and B-O co-doped carbon nanotubes.[57] Besides doping, strategies based on the functionalization of the carbon surface with oxygen rich moieties (-OH, -COOH, C-O-C), as well as amide groups, have been also explored by computational and experimental

methods. Also in this case, the presence of O and N results fundamental in order to bind Li at the surface and reduce the shuttle effect at the cathode.[58]

Going beyond undoped and doped carbon-based matrixes, also transition metal oxides, carbides, nitrides and sulphides have been widely used as electrocatalysts/scaffolds to host the sulfur active material, improve the kinetics of the redox reaction and reduce the shuttle effect through the immobilization of Li-PS.[59–65] Among these materials, M-Xene families are a series of ternary metal carbides, nitrides or carbonitrides with layered structures which allow high electronic conductivity.[66] Their use was proposed in Li/S batteries with a simultaneous N-doping exploiting their crumpled nanosheet morphology.[67] Transition metal compounds have also been investigated as additives to modify surface properties of sulfur compounds. Indeed, their presence in the sulfur compound surface improves the interactions with metal PS, in particular for oxides with redox potential between 2.4V and 3.05 V vs Li^+/Li, which can favour the formation of thiosulfate/polythionate groups[68] Dual functional interconnected fibres embedded with TiO_2/TiN groups, were proposed as free-standing electrodes, showing impressive rate properties and good electrochemical performance.[69]

Open framework materials based on organic assemblies allows the modulation of surface area and porosity, as well as the grafting of functional groups to enhance hydrophilicity. Metal organic frameworks (MOFs) have the presence of a central metal atom that can act as Lewis acid-base sites, strongly interacting with metal PSs.[70] Quasi metal organic framework nanospheres, containing composites of MOFs and metal oxides, are apparently able to promote a large catalytic activity to promote the PS redox chemistry resulting in beneficial effects on the battery capacity stability.[71,72]

As showed by Zheng et al. excellent cycling stability with a capacity retention of 89% after 100 cycles may be obtained by using Ni-containing MOF. The excellent stability is attributed to the synergistic

effects of the porous microstructure, ideal to accommodate Li-PS, and the high binding energies denoting a strong chemical interaction of the transition metal with Li and hindering its diffusion out of the pores.[70] Other studies showed that Ni-MOF matrices not only favour Li-PS retain, but they also improve the kinetics of the redox reaction due to the increased Li diffusion and the reduced polarization potential.[73] Other transition metals MOF have been also proposed and tested with encouraging results, such as Cu-MOF.[74] More complex hybrid architectures based on microporous covalent organic framework (COF) net on mesoporous carbon nanotube (CNT) net has been also used as chemical traps for Li-PS, showing excellent electrochemical performance (capacity retention of 84% after 300 cycles at charge/discharge rate = 2.0 C/2.0 C). In the same work the role of pore size has been investigated by DFT simulations suggesting improved Li-PS absorption occurring in small size pores.[75]

Conductive polymers have also been proposed in Li/S cells thanks to their good electronic conductivity due to the conjugated sequence of double bonds along their chain together with presence of heteroatoms such as O, able to form bonds with PSs upon cycling.[76] An example of 3D structure using conductive polymer is given by the use of poly acrylonitrile (PAN) on the surface of ferroconcrete like network, sulfur-based electrode, to prevent active material dissolution in the electrolyte and enhance its galvanostatic performance.[77] Furthermore the superior activity of polyethylenimine (PEI) to immobilize LiPS in respect to commercial polyvinylidene fluoride (PVDF) has been also demonstrated by DFT calculations.[78]

Towards a suitable electrolyte for reversible Li-sulfur electrochemistry

The development of tailored electrolytes for effective and reversible Li-S batteries has been an inevitable requirement to match the peculiarity of the sulfur redox mechanism (e.g. PS solubility) as well as the weaknesses of any available positive electrode composite electrode and the challenges of the lithium metal counter electrode.

Wang et al. through extensive MD and DFT simulations have been developing useful insights into the mechanism of formation and dissolution of Li-PS. Apparently the formation of Li_2S_n ($1 \leq n \leq 8$) polysuflides upon reduction competes with the formation of large Li-S clusters, particularly for high Li/S ratio. In the presence of low dielectric constant ethereal-based electrolytes (e.g. DME and DOL) small clusters of Li_2S_8, Li_2S_6, and Li_2S_4 show solvation energies larger in respect to dimerization/clusterization energies. Thus, their solubilization by the solvent is highly favourable in the first steps of the reduction, causing depletion of the active sulfur species at the cathode and the poisoning of the anode surface. On the other hand, in the final steps of reduction the clustering is more favourable than the solvent binding, thus explaining the insolubility of Li_2S_2 and Li_2S.[79] Pouya et al. combined Raman and DFT calculations to investigate the Li_2S_4 - Li_2S_2 system. The transition is activated by the clustering of Li_2S_4 species due to the strong electrostatic interactions between Li_2S_4 monomers, which results in energy lowering by arranging the local dipole moments in an anti-parallel fashion.[80] Deep insights into the thermodynamics of PS solvation have been provided by Pascal et al. by MD simulations performed to calculate the thermodynamics of solvation of Li-PS in DMF and diglyme. The authors showed that a significant loss of entropy associated with the PS solvation, compensating the electrostatic interaction, is at the origin of a reduced stabilization of solvated Li-PS, particularly for radical species in polymer-based solvent such as diglyme. This suggests that the use of polyether solvents is a good strategy for the development of future electrolytes.[81] DFT analyses of the thermodynamic stability of $(Li_2S)_n$ ($n \geq 4$) clusters highlight that longer chain species, exhibiting cage-like structures, result to be more structurally stable due to the higher saturation of

dangling bonds with respect to short chain PS. This implies the tendency of small chain PS to cluster. The analysis also shows that the adsorption of PS on different anchoring materials is favored for larger clusters.[82] Vijayakumar et al. showed by DFT calculations that lower-order lithium PS species (i.e. Li_2S_4) favor the clustering or dimerization to form Li_4S_8 (DMSO solvent), while higher-order species (i.e. Li_2S_6 and Li_2S_8) are more stable as monomeric units with lithium at both terminal ends of the chain structure.[83] Kamphaus et al. investigated the structural properties and the thermodynamic stability of long-chain Li-PS Li_2S_6 and Li_2S_8 in two different solvents (DME, DOL) and for different numbers of coordinating Li^+. Beside a strong interaction between the solvent oxygen groups and the Li ion, a dissociation of the Li-PS couple is not observed. This indicates that dissolved Li-PS may exist as locally neutral species through the coordination of additional Li+ and the formation of cluster superstructures within the electrolyte.[84]

According to MD simulations short length PS undergo a clustering within the electrolyte, while a higher solubility is reported for long-chain PS. The stronger $Li^+–S_x^{2-}$ bonding is found to be the primary cause for the low solubility of lower order, while the charge delocalization on the long-chain PS leads to a weakening of the bond and a better solubility in the solvent. The addition of salt (Li–TFSI) weakens the strong $Li^+–S_x^{2-}$ networks due to competing interactions between $TFSI^-$ and PS dianions with Li^+. This competition results in higher Li^+–solvent interactions and increased solubility.[85] Interestingly, other MD simulations showed that while the motion of Li_2S_4 salt in DOL is predominantly vehicular, it is governed by a hopping regime in the more viscous TEGDME solvent. Most importantly, when the two salts are mixed, the presence of the $TFSI^-$ anions does not slow down the diffusion of the PS anion.[86] The diffusion coefficients and conductivities are significantly influenced by the chain length of PS. The conductivity contribution of short chains like S_4^{2-} is lower than that of longer PS chains such as S_6^{2-} or S_8^{2-} even though the diffusion coefficient of S_4^{2-} is higher than that for longer PS chains. The low conductivity of Li_2S_4 can be attributed to its low degree

of dissociation and even to the formation of large clusters in the solution. It is also found that an addition of 1 M LiTFSI into PS solutions considerably reduces the clustering behavior.[87]

In Li/S cells the most common liquid electrolyte is constituted by an ethereal-based solution where a lithium salt is dissolved, typically LiTFSI. In most cases a co-salt is also added, i.e. $LiNO_3$, to promote the reversible lithium stripping/deposition at the negative electrode sides. Other ethers are also largely used like the 1,3-dioxolone (DOL) / 1,2-dimethoxyethane (DME) blend. Ether-based electrolytes are moderating solvating electrolyte (MSE). One of the most relevant challenges is the minimization of the amount of electrolyte in respect to the weight of the positive electrode (or Sulfur): this constraint is essential to achieve practical energy density comparable or above commercial Li-ion systems and minimize the PS dissolution, that is the major drawback in these electrolytes. Qualitatively, the electrolyte to sulfur ratio (E/S) should be below 1 µl/mg to compete with gravimetric performance of standard lithium-ion cells.[88]

Sparingly solvating electrolyte (SSE) configurations can be achieved by tuning the lithium salt concentration towards saturated solutions. In these electrolytes the electrochemical conversion of sulfur can follow different mechanisms, i.e. of quasi-solid or solid-solid conversion.[89] A similar behaviour was also observed in ionic liquid-based electrolyte, e.g. prepared using bis(fluorosulfonyl)imide (FSI^-) anions.[90] Generally speaking the occurrence of a quasi-solid-state mechanism implies the direct conversion of the sulfur active material to the lithium disulphide without the formation of intermediate LiPSs and therefore avoiding all drawbacks originated by the solubility of Li_2S_n.

On the contrary highly solvating electrolyte (HSE) solutions promotes the solubility of Li_2S_n, leading to massive dissolution of PS even in small amounts of electrolyte. In HSE the redox mechanism is therefore fully mediated by the solution and the overall cell configuration resembles a flow cell. In 2015 Liu et al. reported the first concept of HSE electrolyte in Li/S flow batteries, showing high

polarizable DMSO (ε=46.5) to have higher solubility for long and short chain Li_2S_n in respect to DOL/DME solvent mixtures (ε=7): in this electrolyte the saturation concentration of Li_2S_8 exceeds the 14M.[91]

As a last point it is important to recall that remarkable research works to formulate and demonstrate solid electrolytes have been reported in recent years: the research in this field is in its infancy and surely requires large efforts to match performance of liquid electrolytes.[92]

References

(1) Rauh, R. D.; Abraham, K. M.; Pearson, G. F.; Surprenant, J. K.; Brummer, S. B. A Lithium/Dissolved Sulfur Battery with an Organic Electrolyte. *J Electrochem Soc* **1979**, *126* (4), 523–527. https://doi.org/10.1149/1.2129079.

(2) Yamin, H.; Gorenshtein, A.; Penciner, J.; Sternberg, Y.; Peled, E. Lithium Sulfur Battery: Oxidation/Reduction Mechanisms of Polysulfides in THF Solutions. *J Electrochem Soc* **1988**, *135* (5), 1045–1048. https://doi.org/10.1149/1.2095868.

(3) Kolosnitsyn, V. S.; Karaseva, E. V.; Ivanov, A. L. Electrochemistry of a Lithium Electrode in Lithium Polysulfide Solutions. *Russian Journal of Electrochemistry* **2008**, *44* (5), 564–569. https://doi.org/10.1134/S1023193508050091.

(4) Zou, Q.; Liang, Z.; Du, G.-Y.; Liu, C.-Y.; Li, E. Y.; Lu, Y.-C. Cation-Directed Selective Polysulfide Stabilization in Alkali Metal-Sulfur Batteries. *J Am Chem Soc* **2018**, *140* (34), 10740–10748. https://doi.org/10.1021/jacs.8b04536.

(5) He, Q.; Freiberg, A. T. S.; Patel, M. U. M.; Qian, S.; Gasteiger, H. A. Operando Identification of Liquid Intermediates in Lithium–Sulfur Batteries via Transmission UV–Vis Spectroscopy. *J Electrochem Soc* **2020**, *167* (8), 080508. https://doi.org/10.1149/1945-7111/ab8645.

(6) Nelson, J.; Misra, S.; Yang, Y.; Jackson, A.; Liu, Y.; Wang, H.; Dai, H.; Andrews, J. C.; Cui, Y.; Toney, M. F. In Operando X-Ray Diffraction and Transmission X-Ray Microscopy of Lithium Sulfur Batteries. *J Am Chem Soc* **2012**, *134* (14), 6337–6343. https://doi.org/10.1021/ja2121926.

(7) Barchasz, C.; Molton, F.; Duboc, C.; Leprêtre, J.-C.; Patoux, S.; Alloin, F. Lithium/Sulfur Cell Discharge Mechanism: An Original Approach for Intermediate Species Identification. *Anal Chem* **2012**, *84* (9), 3973–3980. https://doi.org/10.1021/ac2032244.

(8) Lu, Y.-C.; He, Q.; Gasteiger, H. A. Probing the Lithium-Sulfur Redox Reactions: A Rotating-Ring Disk Electrode Study. *Journal of Physical Chemistry C* **2014**, *118* (11), 5733–5741. https://doi.org/10.1021/jp500382s.

(9) Wu, H.-L.; Huff, L. A.; Gewirth, A. A. In Situ Raman Spectroscopy of Sulfur Speciation in Lithium-Sulfur Batteries. *ACS Appl Mater Interfaces* **2015**, *7* (3), 1709–1719. https://doi.org/10.1021/am5072942.

(10) Patel, M. U. M.; Dominko, R. Application of in Operando UV/Vis Spectroscopy in Lithium-Sulfur Batteries. *ChemSusChem* **2014**, *7* (8), 2167–2175. https://doi.org/10.1002/cssc.201402215.
(11) Sadd, M.; Agostini, M.; Xiong, S.; Matic, A. Polysulfide Speciation and Migration in Catholyte LithiumÀ Sulfur Cells. **2021**. https://doi.org/10.1002/cphc.202100853.
(12) Sadd, M.; de Angelis, S.; Colding-Jørgensen, S.; Blanchard, D.; Johnsen, R. E.; Sanna, S.; Borisova, E.; Matic, A.; Bowen, J. R. Visualization of Dissolution-Precipitation Processes in Lithium–Sulfur Batteries. *Adv Energy Mater* **2022**, *12* (10). https://doi.org/10.1002/aenm.202103126.
(13) Yan, Y.; Cheng, C.; Zhang, L.; Li, Y.; Lu, J. Deciphering the Reaction Mechanism of Lithium–Sulfur Batteries by In Situ/Operando Synchrotron-Based Characterization Techniques. *Adv Energy Mater* **2019**, *9* (18). https://doi.org/10.1002/aenm.201900148.
(14) Yu, S.-H.; Huang, X.; Schwarz, K.; Huang, R.; Arias, T. A.; Brock, J. D.; Abruña, H. D. Direct Visualization of Sulfur Cathodes: New Insights into Li-S Batteries via Operando X-Ray Based Methods. *Energy Environ Sci* **2018**, *11* (1), 202–210. https://doi.org/10.1039/c7ee02874a.
(15) Cuisinier, M.; Cabelguen, P.-E.; Evers, S.; He, G.; Kolbeck, M.; Garsuch, A.; Bolin, T.; Balasubramanian, M.; Nazar, L. F. Sulfur Speciation in Li-S Batteries Determined by Operando X-Ray Absorption Spectroscopy. *Journal of Physical Chemistry Letters* **2013**, *4* (19), 3227–3232. https://doi.org/10.1021/jz401763d.
(16) Agostini, M.; Xiong, S.; Matic, A.; Hassoun, J. Polysulfide-Containing Glyme-Based Electrolytes for Lithium Sulfur Battery. *Chemistry of Materials* **2015**, *27* (13), 4604–4611. https://doi.org/10.1021/acs.chemmater.5b00896.
(17) Xiong, S.; Xie, K.; Diao, Y.; Hong, X. Characterization of the Solid Electrolyte Interphase on Lithium Anode for Preventing the Shuttle Mechanism in Lithium-Sulfur Batteries. *J Power Sources* **2014**, *246*, 840–845. https://doi.org/10.1016/j.jpowsour.2013.08.041.
(18) Zhang, S. S. Effect of Discharge Cutoff Voltage on Reversibility of Lithium/Sulfur Batteries with LiNO3-Contained Electrolyte. *J Electrochem Soc* **2012**, *159* (7), A920–A923. https://doi.org/10.1149/2.002207jes.
(19) Liang, X.; Wen, Z.; Liu, Y.; Wu, M.; Jin, J.; Zhang, H.; Wu, X. Improved Cycling Performances of Lithium Sulfur Batteries with LiNO <inf>3</Inf>-Modified Electrolyte. *J Power Sources* **2011**, *196* (22), 9839–9843. https://doi.org/10.1016/j.jpowsour.2011.08.027.
(20) Aurbach, D.; Pollak, E.; Elazari, R.; Salitra, G.; Kelley, C. S.; Affinito, J. On the Surface Chemical Aspects of Very High Energy Density, Rechargeable Li–Sulfur Batteries. *J Electrochem Soc* **2009**, *156* (8), A694. https://doi.org/10.1149/1.3148721.
(21) Feng, S.; Fu, Z.-H.; Chen, X.; Zhang, Q. A Review on Theoretical Models for Lithium–Sulfur Battery Cathodes. *InfoMat* **2022**, *4* (3). https://doi.org/10.1002/inf2.12304.
(22) Chen, X.; Hou, T.; Persson, K. A.; Zhang, Q. Combining Theory and Experiment in Lithium–Sulfur Batteries: Current Progress and Future Perspectives. *Materials Today* **2019**, *22*, 142–158. https://doi.org/10.1016/j.mattod.2018.04.007.
(23) Zhang, Z.-W.; Peng, H.-J.; Zhao, M.; Huang, J.-Q. Heterogeneous/Homogeneous Mediators for High-Energy-Density Lithium–Sulfur Batteries: Progress and Prospects. *Adv Funct Mater* **2018**, *28* (38). https://doi.org/10.1002/adfm.201707536.
(24) Chen, C.-Y.; Peng, H.-J.; Hou, T.-Z.; Zhai, P.-Y.; Li, B.-Q.; Tang, C.; Zhu, W.; Huang, J.-Q.; Zhang, Q. A Quinonoid-Imine-Enriched Nanostructured Polymer Mediator for Lithium–Sulfur Batteries. *Advanced Materials* **2017**, *29* (23). https://doi.org/10.1002/adma.201606802.
(25) Pang, Q.; Kundu, D.; Cuisinier, M.; Nazar, L. F. Surface-Enhanced Redox Chemistry of Polysulphides on a Metallic and Polar Host for Lithium-Sulphur Batteries. *Nat Commun* **2014**, *5*. https://doi.org/10.1038/ncomms5759.

(26) Li, J.; Qu, Y.; Chen, C.; Zhang, X.; Shao, M. Theoretical Investigation on Lithium Polysulfide Adsorption and Conversion for High-Performance Li-S Batteries. *Nanoscale* **2021**, *13* (1), 15–35. https://doi.org/10.1039/d0nr06732f.

(27) Du, Z.; Chen, X.; Hu, W.; Chuang, C.; Xie, S.; Hu, A.; Yan, W.; Kong, X.; Wu, X.; Ji, H.; Ji, H.; Wan, L.-J. Cobalt in Nitrogen-Doped Graphene as Single-Atom Catalyst for High-Sulfur Content Lithium-Sulfur Batteries. *J Am Chem Soc* **2019**, *141* (9), 3977–3985. https://doi.org/10.1021/jacs.8b12973.

(28) Zhou, G.; Zhao, S.; Wang, T.; Yang, S.-Z.; Johannessen, B.; Chen, H.; Liu, C.; Ye, Y.; Wu, Y.; Peng, Y.; Liu, C.; San, ∇; Jiang, P.; Zhang, Q.; Cui, Y. Theoretical Calculation Guided Design of Single-Atom Catalysts toward Fast Kinetic and Long-Life Li−S Batteries. *Nano Lett* **2020**, *20* (2), 1252–1261. https://doi.org/10.1021/acs.nanolett.9b04719.

(29) Wang, C.; Sun, L.; Li, K.; Wu, Z.; Zhang, F.; Wang, L. Unravel the Catalytic Effect of Two-Dimensional Metal Sulfides on Polysulfide Conversions for Lithium-Sulfur Batteries. *ACS Appl Mater Interfaces* **2020**, *12* (39), 43560–43567. https://doi.org/10.1021/acsami.0c09567.

(30) Shao, Q.; Lu, P.; Xu, L.; Guo, D.; Gao, J.; Wu, Z.-S.; Chen, J. Rational Design of MoS<inf>2</Inf> Nanosheets Decorated on Mesoporous Hollow Carbon Spheres as a Dual-Functional Accelerator in Sulfur Cathode for Advanced Pouch-Type Li–S Batteries. *Journal of Energy Chemistry* **2020**, *51*, 262–271. https://doi.org/10.1016/j.jechem.2020.03.035.

(31) Zhou, G.; Tian, H.; Jin, Y.; Tao, X.; Liu, B.; Zhang, R.; Seh, Z. W.; Zhuo, D.; Liu, Y.; Sun, J.; Zhang, Q.; Cui, Y. Catalytic Oxidation of Li<inf>2</Inf>S on the Surface of Metal Sulfides for Li-S Batteries. *Proc Natl Acad Sci U S A* **2017**, *114* (5), 840–845. https://doi.org/10.1073/pnas.1615837114.

(32) Hu, G.; Xu, C.; Sun, Z.; Wang, S.; Cheng, H.-M.; Li, F.; Ren, W. 3D Graphene-Foam-Reduced-Graphene-Oxide Hybrid Nested Hierarchical Networks for High-Performance Li-S Batteries. *Advanced Materials* **2016**, *28* (8), 1603–1609. https://doi.org/10.1002/adma.201504765.

(33) Nitze, F.; Agostini, M.; Lundin, F.; Palmqvist, A. E. C.; Matic, A. A Binder-Free Sulfur/Reduced Graphene Oxide Aerogel as High Performance Electrode Materials for Lithium Sulfur Batteries. *Sci Rep* **2016**, *6* (1), 39615. https://doi.org/10.1038/srep39615.

(34) Hou, T.-Z.; Chen, X.; Peng, H.-J.; Huang, J.-Q.; Li, B.-Q.; Zhang, Q.; Li, B. Design Principles for Heteroatom-Doped Nanocarbon to Achieve Strong Anchoring of Polysulfides for Lithium–Sulfur Batteries. *Small* **2016**, 3283–3291. https://doi.org/10.1002/smll.201600809.

(35) Tikekar, M. D.; Choudhury, S.; Tu, Z.; Archer, L. A. Design Principles for Electrolytes and Interfaces for Stable Lithium-Metal Batteries. *Nat Energy* **2016**, *1*, 16114. https://doi.org/10.1038/nenergy.2016.114.

(36) Zheng, Y.; Li, H.; Yuan, H.; Fan, H.; Li, W.; Zhang, J. Understanding the Anchoring Effect of Graphene, BN, C <inf>2</Inf> N and C <inf>3</Inf> N <inf>4</Inf> Monolayers for Lithium−polysulfides in Li−S Batteries. *Appl Surf Sci* **2018**, *434*, 596–603. https://doi.org/10.1016/j.apsusc.2017.10.230.

(37) Hou, T.-Z.; Xu, W.-T.; Chen, X.; Peng, H.-J.; Huang, J.-Q.; Zhang, Q. Lithium Bond Chemistry in Lithium–Sulfur Batteries. *Angewandte Chemie - International Edition* **2017**, *56* (28), 8178–8182. https://doi.org/10.1002/anie.201704324.

(38) Chen, X.; Peng, H.-J.; Zhang, R.; Hou, T.-Z.; Huang, J.-Q.; Li, B.; Zhang, Q. An Analogous Periodic Law for Strong Anchoring of Polysulfides on Polar Hosts in Lithium Sulfur Batteries: S- or Li-Binding on First-Row Transition-Metal Sulfides? *ACS Energy Lett* **2017**, *2* (4), 795–801. https://doi.org/10.1021/acsenergylett.7b00164.

(39) Tao, X.; Wan, J.; Liu, C.; Wang, H.; Yao, H.; Zheng, G.; Seh, Z. W.; Cai, Q.; Li, W.; Zhou, G.; Zu, C.; Cui, Y. Balancing Surface Adsorption and Diffusion of Lithium-Polysulfides on Nonconductive Oxides for Lithium-Sulfur Battery Design. *Nat Commun* **2016**, *7*. https://doi.org/10.1038/ncomms11203.

(40) Schuster, J.; He, G.; Mandlmeier, B.; Yim, T.; Lee, K. T.; Bein, T.; Nazar, L. F. Spherical Ordered Mesoporous Carbon Nanoparticles with High Porosity for Lithium-Sulfur Batteries. *Angewandte Chemie - International Edition* **2012**, *51* (15), 3591–3595. https://doi.org/10.1002/anie.201107817.

(41) He, Q.; Liao, X.; Xia, L.; Li, Z.; Wang, H.; Zhao, Y.; Truhlar, D. G. Accurate Binding Energies for Lithium Polysulfides and Assessment of Density Functionals for Lithium-Sulfur Battery Research. *Journal of Physical Chemistry C* **2019**, *123* (34), 20737–20747. https://doi.org/10.1021/acs.jpcc.9b05235.

(42) Dörfler, S.; Strubel, P.; Jaumann, T.; Troschke, E.; Hippauf, F.; Kensy, C.; Schökel, A.; Althues, H.; Giebeler, L.; Oswald, S.; Oswald, S.; Kaskel, S. On the Mechanistic Role of Nitrogen-Doped Carbon Cathodes in Lithium-Sulfur Batteries with Low Electrolyte Weight Portion. *Nano Energy* **2018**, *54*, 116–128. https://doi.org/10.1016/j.nanoen.2018.09.065.

(43) Song, J.; Xu, T.; Gordin, M. L.; Zhu, P.; Lv, D.; Jiang, Y.-B.; Chen, Y.; Duan, Y.; Wang, D. Nitrogen-Doped Mesoporous Carbon Promoted Chemical Adsorption of Sulfur and Fabrication of High-Areal-Capacity Sulfur Cathode with Exceptional Cycling Stability for Lithium-Sulfur Batteries. *Adv Funct Mater* **2014**, *24* (9), 1243–1250. https://doi.org/10.1002/adfm.201302631.

(44) Ji, L.; Rao, M.; Zheng, H.; Zhang, L.; Li, Y.; Duan, W.; Guo, J.; Cairns, E. J.; Zhang, Y. Graphene Oxide as a Sulfur Immobilizer in High Performance Lithium/Sulfur Cells. *J Am Chem Soc* **2011**, *133* (46), 18522–18525. https://doi.org/10.1021/ja206955k.

(45) Sun, J.; Hwang, J.-Y.; Jankowski, P.; Xiao, L.; Sanchez, J. S.; Xia, Z.; Lee, S.; Talyzin, A. V.; Matic, A.; Palermo, V.; Sun, Y.-K.; Agostini, M. Critical Role of Functional Groups Containing N, S, and O on Graphene Surface for Stable and Fast Charging Li-S Batteries. *Small* **2021**. https://doi.org/10.1002/smll.202007242.

(46) Li, W.; Ye, Y.; Qian, J.; Xing, Y.; Qu, W.; Zhang, N.; Li, L.; Wu, F.; Chen, R. Oxygenated Nitrogen-Doped Microporous Nanocarbon as a Permselective Interlayer for Ultrastable Lithium-Sulfur Batteries. *ChemElectroChem* **2019**, *6* (4), 1094–1100. https://doi.org/10.1002/celc.201801525.

(47) Wang, Y.; Huang, J.; Chen, X.; Wang, L.; Ye, Z. Powder Metallurgy Template Growth of 3D N-Doped Graphene Foam as Binder-Free Cathode for High-Performance Lithium/Sulfur Battery. *Carbon N Y* **2018**, *137*, 368–378. https://doi.org/10.1016/j.carbon.2018.05.048.

(48) Zhou, G.; Zhao, Y.; Manthiram, A. Dual-Confined Flexible Sulfur Cathodes Encapsulated in Nitrogen-Doped Double-Shelled Hollow Carbon Spheres and Wrapped with Graphene for Li-S Batteries. *Adv Energy Mater* **2015**, *5* (9). https://doi.org/10.1002/aenm.201402263.

(49) Shu, Y.; Li, X.; Ye, J.; Gao, W.; Cheng, S.; Zhang, X.; Ma, L.; Ding, Y. Improved Performance and Immobilizing Mechanism of N-Doping Carbon Aerogel with Net Channel via Long-Chain Directing for Lithium–Sulfur Battery. *Energy Technology* **2020**, *8* (3). https://doi.org/10.1002/ente.201901057.

(50) Ai, W.; Li, J.; Du, Z.; Zou, C.; Du, H.; Xu, X.; Chen, Y.; Zhang, H.; Zhao, J.; Li, C.; Huang, W.; Yu, T. Dual Confinement of Polysulfides in Boron-Doped Porous Carbon Sphere/Graphene Hybrid for Advanced Li-S Batteries. *Nano Res* **2018**, *11* (9), 4562–4573. https://doi.org/10.1007/s12274-018-2036-6.

(51) Ma, X.; Ning, G.; Wang, Y.; Song, X.; Xiao, Z.; Hou, L.; Yang, W.; Gao, J.; Li, Y. S-Doped Mesoporous Graphene Microspheres: A High Performance Reservoir Material for Li–S Batteries. *Electrochim Acta* **2018**, *269*, 83–92. https://doi.org/10.1016/j.electacta.2018.02.163.

(52) Balach, J.; Singh, H. K.; Gomoll, S.; Jaumann, T.; Klose, M.; Oswald, S.; Richter, M.; Eckert, J.; Giebeler, L. Synergistically Enhanced Polysulfide Chemisorption Using a Flexible Hybrid Separator with N and S Dual-Doped Mesoporous Carbon Coating for Advanced Lithium-Sulfur Batteries. *ACS Appl Mater Interfaces* **2016**, *8* (23), 14586–14595. https://doi.org/10.1021/acsami.6b03642.

(53) Zhou, G.; Paek, E.; Hwang, G. S.; Manthiram, A. Long-Life Li/Polysulphide Batteries with High Sulphur Loading Enabled by Lightweight Three-Dimensional Nitrogen/Sulphur-Codoped Graphene Sponge. *Nat Commun* **2015**, *6*. https://doi.org/10.1038/ncomms8760.

(54) Pang, Q.; Tang, J.; Huang, H.; Liang, X.; Hart, C.; Tam, K. C.; Nazar, L. F. A Nitrogen and Sulfur Dual-Doped Carbon Derived from Polyrhodanine@Cellulose for Advanced Lithium-Sulfur Batteries. *Advanced Materials* **2015**, *27* (39), 6021–6028. https://doi.org/10.1002/adma.201502467.

(55) Ai, W.; Luo, Z.; Jiang, J.; Zhu, J.; Du, Z.; Fan, Z.; Xie, L.; Zhang, H.; Huang, W.; Yu, T. Nitrogen and Sulfur Codoped Graphene: Multifunctional Electrode Materials for High-Performance LI-Ion Batteries and Oxygen Reduction Reaction. *Advanced Materials* **2014**, *26* (35), 6186–6192. https://doi.org/10.1002/adma.201401427.

(56) Gu, X.; Tong, C.-J.; Lai, C.; Qiu, J.; Huang, X.; Yang, W.; Wen, B.; Liu, L.-M.; Hou, Y.; Zhang, S. A Porous Nitrogen and Phosphorous Dual Doped Graphene Blocking Layer for High Performance Li-S Batteries. *J Mater Chem A Mater* **2015**, *3* (32), 16670–16678. https://doi.org/10.1039/c5ta04255k.

(57) Jin, C.; Zhang, W.; Zhuang, Z.; Wang, J.; Huang, H.; Gan, Y.; Xia, Y.; Liang, C.; Zhang, J.; Tao, X. Enhanced Sulfide Chemisorption Using Boron and Oxygen Dually Doped Multi-Walled Carbon Nanotubes for Advanced Lithium-Sulfur Batteries. *J Mater Chem A Mater* **2017**, *5* (2), 632–640. https://doi.org/10.1039/c6ta07620c.

(58) Maihom, T.; Kaewruang, S.; Phattharasupakun, N.; Chiochan, P.; Limtrakul, J.; Sawangphruk, M. Lithium Bond Impact on Lithium Polysulfide Adsorption with Functionalized Carbon Fiber Paper Interlayers for Lithium-Sulfur Batteries. *Journal of Physical Chemistry C* **2018**, *122* (13), 7033–7040. https://doi.org/10.1021/acs.jpcc.7b09392.

(59) Sun, M.; Wang, Z.; Li, X.; Li, H.; Jia, H.; Xue, X.; Jin, M.; Li, J.; Xie, Y.; Feng, M. Rational Understanding of the Catalytic Mechanism of Molybdenum Carbide in Polysulfide Conversion in Lithium-Sulfur Batteries. *J Mater Chem A Mater* **2020**, *8* (23), 11818–11823. https://doi.org/10.1039/d0ta01217c.

(60) Yu, J.; Xiao, J.; Li, A.; Yang, Z.; Zeng, L.; Zhang, Q.; Zhu, Y.; Guo, L. Enhanced Multiple Anchoring and Catalytic Conversion of Polysulfides by Amorphous MoS<inf>3</Inf> Nanoboxes for High-Performance Li-S Batteries. *Angewandte Chemie - International Edition* **2020**, *59* (31), 13071–13078. https://doi.org/10.1002/anie.202004914.

(61) Xiao, K.; Chen, Z.; Liu, Z.; Zhang, L.; Cai, X.; Song, C.; Fan, Z.; Chen, X.; Liu, J.; Shen, Z. X. N-Doped Carbon Sheets Arrays Embedded with CoP Nanoparticles as High-Performance Cathode for Li-S Batteries via Triple Synergistic Effects. *J Power Sources* **2020**, *455*. https://doi.org/10.1016/j.jpowsour.2020.227959.

(62) Li, Z.; Zhang, J.; Lou, X. W. Hollow Carbon Nanofibers Filled with MnO<inf>2</Inf> Nanosheets as Efficient Sulfur Hosts for Lithium-Sulfur Batteries. *Angewandte Chemie - International Edition* **2015**, *54* (44), 12886–12890. https://doi.org/10.1002/anie.201506972.

(63) Liang, X.; Hart, C.; Pang, Q.; Garsuch, A.; Weiss, T.; Nazar, L. F. A Highly Efficient Polysulfide Mediator for Lithium-Sulfur Batteries. *Nat Commun* **2015**, *6*. https://doi.org/10.1038/ncomms6682.

(64) Seh, Z. W.; Li, W.; Cha, J. J.; Zheng, G.; Yang, Y.; McDowell, M. T.; Hsu, P.-C.; Cui, Y. Sulphur-TiO 2 Yolk-Shell Nanoarchitecture with Internal Void Space for Long-Cycle Lithium-Sulphur Batteries. *Nat Commun* **2013**, *4*. https://doi.org/10.1038/ncomms2327.

(65) Shen, Z.; Zhang, Z.; Li, M.; Yuan, Y.; Zhao, Y.; Zhang, S.; Zhong, C.; Zhu, J.; Lu, J.; Zhang, H. Rational Design of a Ni<inf>3</Inf>N<inf>0.85</Inf>Electrocatalyst to Accelerate Polysulfide Conversion in Lithium-Sulfur Batteries. *ACS Nano* **2020**, *14* (6), 6673–6682. https://doi.org/10.1021/acsnano.9b09371.

(66) Liu, Y.-H.; Wang, C.-Y.; Yang, S.-L.; Cao, F.-F.; Ye, H. 3D MXene Architectures as Sulfur Hosts for High-Performance Lithium-Sulfur Batteries. *Journal of Energy Chemistry* **2022**, *66*, 429–439. https://doi.org/10.1016/j.jechem.2021.08.040.

(67) Bao, W.; Liu, L.; Wang, C.; Choi, S.; Wang, D.; Wang, G. Facile Synthesis of Crumpled Nitrogen-Doped MXene Nanosheets as a New Sulfur Host for Lithium–Sulfur Batteries. *Adv Energy Mater* **2018**, *8* (13). https://doi.org/10.1002/aenm.201702485.

(68) Liang, X.; Kwok, C. Y.; Lodi-Marzano, F.; Pang, Q.; Cuisinier, M.; Huang, H.; Hart, C. J.; Houtarde, D.; Kaup, K.; Sommer, H.; Janek, J.; Nazar, L. F. Tuning Transition Metal Oxide-Sulfur Interactions for Long Life Lithium Sulfur Batteries: The "Goldilocks" Principle. *Adv Energy Mater* **2016**, *6* (6). https://doi.org/10.1002/aenm.201501636.

(69) Xue, P.; Zhu, K.; Gong, W.; Pu, J.; Li, X.; Guo, C.; Wu, L.; Wang, R.; Li, H.; Sun, J.; Zhang, Q.; Yao, Y. "One Stone Two Birds" Design for Dual-Functional TiO<inf>2</Inf>-TiN Heterostructures Enabled Dendrite-Free and Kinetics-Enhanced Lithium–Sulfur Batteries. *Adv Energy Mater* **2022**, *12* (18). https://doi.org/10.1002/aenm.202200308.

(70) Zheng, J.; Tian, J.; Wu, D.; Gu, M.; Xu, W.; Wang, C.; Gao, F.; Engelhard, M. H.; Zhang, J.-G.; Liu, J.; Liu, J.; Xiao, J. Lewis Acid-Base Interactions between Polysulfides and Metal Organic Framework in Lithium Sulfur Batteries. *Nano Lett* **2014**, *14* (5), 2345–2352. https://doi.org/10.1021/nl404721h.

(71) Luo, D.; Li, C.; Zhang, Y.; Ma, Q.; Ma, C.; Nie, Y.; Li, M.; Weng, X.; Huang, R.; Zhao, Y.; Wang, X.; Chen, Z. Design of Quasi-MOF Nanospheres as a Dynamic Electrocatalyst toward Accelerated Sulfur Reduction Reaction for High-Performance Lithium–Sulfur Batteries. *Advanced Materials* **2022**, *34* (2). https://doi.org/10.1002/adma.202105541.

(72) Bai, S.; Liu, X.; Zhu, K.; Wu, S.; Zhou, H. Metal-Organic Framework-Based Separator for Lithium-Sulfur Batteries. *Nat Energy* **2016**, *1* (7). https://doi.org/10.1038/nenergy.2016.94.

(73) Yang, Y.; Wang, Z.; Jiang, T.; Dong, C.; Mao, Z.; Lu, C.; Sun, W.; Sun, K. A Heterogenized Ni-Doped Zeolitic Imidazolate Framework to Guide Efficient Trapping and Catalytic Conversion of Polysulfides for Greatly Improved Lithium-Sulfur Batteries. *J Mater Chem A Mater* **2018**, *6* (28), 13593–13598. https://doi.org/10.1039/c8ta05176c.

(74) Wang, Z.; Wang, B.; Yang, Y.; Cui, Y.; Wang, Z.; Chen, B.; Qian, G. Mixed-Metal-Organic Framework with Effective Lewis Acidic Sites for Sulfur Confinement in High-Performance Lithium-Sulfur Batteries. *ACS Appl Mater Interfaces* **2015**, *7* (37), 20999–21004. https://doi.org/10.1021/acsami.5b07024.

(75) Yoo, J.; Cho, S.-J.; Jung, G. Y.; Kim, S. H.; Choi, K.-H.; Kim, J.-H.; Lee, C. K.; Kwak, S. K.; Lee, S.-Y. COF-Net on CNT-Net as a Molecularly Designed, Hierarchical Porous Chemical Trap for Polysulfides in Lithium-Sulfur Batteries. *Nano Lett* **2016**, *16* (5), 3292–3300. https://doi.org/10.1021/acs.nanolett.6b00870.

(76) Yang, Y.; Yu, G.; Cha, J. J.; Wu, H.; Vosgueritchian, M.; Yao, Y.; Bao, Z.; Cui, Y. Improving the Performance of Lithium-Sulfur Batteries by Conductive Polymer Coating. *ACS Nano* **2011**, *5* (11), 9187–9193. https://doi.org/10.1021/nn203436j.

(77) Yan, M.; Chen, H.; Yu, Y.; Zhao, H.; Li, C.-F.; Hu, Z.-Y.; Wu, P.; Chen, L.; Wang, H.; Peng, D.; Li, Y.; Su, B.-L. 3D Ferroconcrete-Like Aminated Carbon Nanotubes Network Anchoring Sulfur for Advanced Lithium–Sulfur Battery. *Adv Energy Mater* **2018**, *8* (25). https://doi.org/10.1002/aenm.201801066.

(78) Liao, J.; Liu, Z.; Liu, X.; Ye, Z. Water-Soluble Linear Poly(Ethylenimine) as a Superior Bifunctional Binder for Lithium-Sulfur Batteries of Improved Cell Performance. *Journal of Physical Chemistry C* **2018**, *122* (45), 25917–25929. https://doi.org/10.1021/acs.jpcc.8b09378.

(79) Wang, B.; Alhassan, S. M.; Pantelides, S. T. Formation of Large Polysulfide Complexes during the Lithium-Sulfur Battery Discharge. *Phys Rev Appl* **2014**, *2* (3). https://doi.org/10.1103/PhysRevApplied.2.034004.

(80) Partovi-Azar, P.; Kühne, T. D.; Kaghazchi, P. Evidence for the Existence of Li<inf>2</Inf>S<inf>2</Inf> Clusters in Lithium-Sulfur Batteries: Ab Initio Raman Spectroscopy Simulation. *Physical Chemistry Chemical Physics* **2015**, *17* (34), 22009–22014. https://doi.org/10.1039/c5cp02781k.

(81) Pascal, T. A.; Wujcik, K. H.; Wang, D. R.; Balsara, N. P.; Prendergast, D. Thermodynamic Origins of the Solvent-Dependent Stability of Lithium Polysulfides from First Principles. *Physical Chemistry Chemical Physics* **2017**, *19* (2), 1441–1448. https://doi.org/10.1039/c6cp06889h.
(82) Yu, T.; Li, F.; Liu, C.; Zhang, S.; Xu, H.; Yang, G. Understanding the Role of Lithium Sulfide Clusters in Lithium-Sulfur Batteries. *J Mater Chem A Mater* **2017**, *5* (19), 9293–9298. https://doi.org/10.1039/c7ta01006k.
(83) Vijayakumar, M.; Govind, N.; Walter, E.; Burton, S. D.; Shukla, A.; Devaraj, A.; Xiao, J.; Liu, J.; Wang, C.; Karim, A.; Karim, A.; Thevuthasan, S. Molecular Structure and Stability of Dissolved Lithium Polysulfide Species. *Physical Chemistry Chemical Physics* **2014**, *16* (22), 10923–10932. https://doi.org/10.1039/c4cp00889h.
(84) Kamphaus, E. P.; Balbuena, P. B. First-Principles Investigation of Lithium Polysulfide Structure and Behavior in Solution. *Journal of Physical Chemistry C* **2017**, *121* (39), 21105–21117. https://doi.org/10.1021/acs.jpcc.7b04822.
(85) Rajput, N. N.; Murugesan, V.; Shin, Y.; Han, K. S.; Lau, K. C.; Chen, J.; Liu, J.; Curtiss, L. A.; Mueller, K. T.; Persson, K. A. Elucidating the Solvation Structure and Dynamics of Lithium Polysulfides Resulting from Competitive Salt and Solvent Interactions. *Chemistry of Materials* **2017**, *29* (8), 3375–3379. https://doi.org/10.1021/acs.chemmater.7b00068.
(86) Osella, S.; Minoia, A.; Quarti, C.; Cornil, J.; Lazzaroni, R.; Goffin, A.-L.; Guillaume, M.; Beljonne, D. Modelling Coupled Ion Motion in Electrolyte Solutions for Lithium-Sulfur Batteries. *Batter Supercaps* **2019**, *2* (5), 473–481. https://doi.org/10.1002/batt.201800150.
(87) Park, C.; Ronneburg, A.; Risse, S.; Ballauff, M.; Kanduč, M.; Dzubiella, J. Structural and Transport Properties of Li/S Battery Electrolytes: Role of the Polysulfide Species. *Journal of Physical Chemistry C* **2019**, *123* (16), 10167–10177. https://doi.org/10.1021/acs.jpcc.8b10175.
(88) Agostini, M.; Hwang, J.-Y.; Kim, H. M.; Bruni, P.; Brutti, S.; Croce, F.; Matic, A.; Sun, Y.-K. Minimizing the Electrolyte Volume in Li–S Batteries: A Step Forward to High Gravimetric Energy Density. *Adv Energy Mater* **2018**, *8* (26). https://doi.org/10.1002/aenm.201801560.
(89) Pang, Q.; Shyamsunder, A.; Narayanan, B.; Kwok, C. Y.; Curtiss, L. A.; Nazar, L. F. Tuning the Electrolyte Network Structure to Invoke Quasi-Solid State Sulfur Conversion and Suppress Lithium Dendrite Formation in Li–S Batteries. *Nat Energy* **2018**, *3* (9), 783–791. https://doi.org/10.1038/s41560-018-0214-0.
(90) Markevich, E.; Salitra, G.; Talyosef, Y.; Chesneau, F.; Aurbach, D. Review—On the Mechanism of Quasi-Solid-State Lithiation of Sulfur Encapsulated in Microporous Carbons: Is the Existence of Small Sulfur Molecules Necessary? *J Electrochem Soc* **2017**, *164* (1), A6244–A6253. https://doi.org/10.1149/2.0391701jes.
(91) Pan, H.; Wei, X.; Henderson, W. A.; Shao, Y.; Chen, J.; Bhattacharya, P.; Xiao, J.; Liu, J. On the Way Toward Understanding Solution Chemistry of Lithium Polysulfides for High Energy Li-S Redox Flow Batteries. *Adv Energy Mater* **2015**, *5* (16). https://doi.org/10.1002/aenm.201500113.
(92) Wang, H.; Cao, X.; Liu, W.; Sun, X. Research Progress of the Solid State Lithium-Sulfur Batteries. *Front Energy Res* **2019**, *7*. https://doi.org/10.3389/fenrg.2019.00112.
